# Are two naïve and distributed heads better than one? Factors influencing the performance of teams in a challenging real-time task

**DOI:** 10.3389/fpsyg.2023.1042710

**Published:** 2023-05-12

**Authors:** Matthew D. Blanchard, Sabina Kleitman, Eugene Aidman

**Affiliations:** ^1^School of Psychology, The University of Sydney, Darlington, NSW, Australia; ^2^Defence Science and Technology Group, Land Division, Edinburgh, SA, Australia

**Keywords:** two heads better than one, collective decisions, dyads, team performance, communication quality, driving simulation

## Abstract

**Introduction:**

Collective decisions in dynamic tasks can be influenced by multiple factors, including the operational conditions, quality and quantity of communication, and individual differences. These factors may influence whether two heads perform better than one. This study examined the “two heads are better than one” effect (2HBT1) in distributed two-person driver-navigator teams with asymmetrical roles performing a challenging simulated driving task. We also examined the influence of communication quality and quantity on team performance under different operational conditions. In addition to traditional measures of communication volume (duration and speaking turns), patterns of communication quality (optimality of timing and accuracy of instructions) were captured.

**Methods:**

Participants completed a simulated driving task under two operational conditions (normal and fog) either as individual drivers (*N* = 134; 87 females, mean age = 19.80, SD = 3.35) or two-person teams (driver and navigator; *N* = 80; 109 females, mean age = 19.70, SD = 4.69). The normal condition was characterized by high visibility for both driver and navigator. The fog condition was characterized by reduced visibility for the driver but not for the navigator. Participants were also measured on a range of cognitive and personality constructs.

**Results:**

Teams had fewer collisions than individuals during normal conditions but not during fog conditions when teams had an informational advantage over individuals. Furthermore, teams drove slower than individuals during fog conditions but not during normal conditions. Communication that was poorly timed and/or inaccurate was a positive predictor of accuracy (i.e., collisions) during the normal condition and communication that was well timed and accurate was a negative predictor of speed during the fog condition. Our novel measure of communication quality (i.e., content of communication) was a stronger predictor of accuracy, but volume of communication was a stronger predictor of time (i.e., speed).

**Discussion:**

Results indicate when team performance thrives and succumbs compared with individual performance and informs theory about the 2HBT1 effect and team communication.

## Introduction

Technological advances and situational necessities, like the current pandemic, have increased the ability of geographically distributed individuals to work together (Bell and Kozlowski, 2002), particularly in dynamic environments which are characterized by high time and cognitive resources demands, ambiguous and rapidly changing information, and multiple connected decisions. Real-world scenarios, especially those involving distributed teams performing high-stakes, mission-critical tasks are often characterized by a deliberate division of labor across team members who perform different roles to achieve mission objectives. In these distinct roles, team members typically have access to different information about the operating environment. The pooling of information from these diverse perspectives confers performance advantages. Examples include air traffic controllers and pilots, forward observers guiding artillery or air strikes in combat zones, and operations rooms feeding information to emergency services, such as police or firefighters. Despite the growing need to study teamwork in dynamic environments, many studies have focused on static tasks. These studies examined the “two heads are better than one” effect (2HBT1; e.g., Sniezek and Henry, 1989; Bahrami et al., 2010; Koriat, 2015) and demonstrated that it depends on the characteristics of the task (e.g., Koriat, 2012, 2015). However, there is a scarcity of research that has examined this effect using dynamic tasks, and to our knowledge, none examined it using distributed teams with asymmetrical roles or under different operational conditions (i.e., task characteristics). Given that the use of distributed teams is continuing to increase, especially during the pandemic, it is critical to understand the relationship between the 2HBT1 effect for asymmetrical teams and the characteristics of a dynamic task with varying operational conditions.

When working in a team, communication is an essential process that teams use to share and process information (Hinsz et al., 1997). Effective communication is associated with fewer performance errors across a broad range of tasks (e.g., Foushee, 1984; Donchin et al., 1995; Helmreich et al., 1999; Christensen et al., 2000; Kanki et al., 2010). As Marlow et al. (2018) highlighted in their review, no studies have yet examined the relationship between communication and asymmetrical team performance under different operational conditions. Moreover, according to Marlow and colleagues, assessments of the quality of communication typically use self-report questionnaires administered post-task. This type of measurement suffers from several response distortions (e.g., Sackett, 1979; Arnold and Feldman, 1981). To address these limitations, we developed and used a novel methodology to assess the quality of communication (i.e., the accuracy and timeliness of information) shared between team members while they performed together during a dynamic task with different operational conditions. Our more objective measure was based on the observation of actual team dynamics rather than recollections and self-reports, and it allowed us to examine the relationship between the quality of communication and two common metrics of team performance (accuracy and time). If the 2HBT1 effect depends on the operational conditions of a dynamic task, then the relationship between communication and performance may also depend on the operational condition. Our research addresses this hypothesis.

*The present study*: we compared the performance of individuals and naïve, asymmetrical, distributed teams during two operational conditions within the same dynamic task. We also developed a novel method of measuring communication dynamics to examine their relationship with team performance. We used naïve (also known as *ad hoc*) teams, whose members had no prior experience working together. Such teams are commonly used in business and industry (Devine et al., 1999; Sundstrom et al., 2000) and have greater potential for failure compared with experienced teams. It is important to understand their performance outcomes and the role of communication as a predictor of performance.

### Are 2HBT1 under different operational conditions?

The literature is replete with studies demonstrating that two heads are more accurate than one on tasks with *static* environments (e.g., Hill, 1982; Sniezek and Henry, 1989; Bahrami et al., 2010; Laughlin, 2011; Koriat, 2015). This effect, however, depends on the characteristics of the task. For example, Koriat (2012, 2015) found that two heads are better than one for non-misleading questions on cognitive tests, but worse for misleading test questions. These studies have largely examined the 2HBT1 effect by assigning members to symmetrical teams where each member performs the same role and is exposed to the same conditions/information (Hill, 1982; Sniezek and Henry, 1989; Laughlin, 2011). For example, Koriat (2015) had two person teams complete a general knowledge test together and both members were exposed to the same questions under the same conditions. However, several studies have introduced asymmetry by assigning members to the same role but exposing them to different conditions/information (Bahrami et al., 2010, 2012; Mahmoodi et al., 2015; Pescetelli et al., 2016). For example, Bahrami et al. (2010) had teams complete a visual perception task where participants were briefly shown a pattern comprised of smaller circles and one circle had a higher contrast than the others. Participants had to identify which circle in the pattern had higher contrast. During some trials, one team member had noise added to the pattern which conferred an informational advantage to the other member. Bahrami et al. found that when team members received the same or differing information a 2HBT1 effect was observed. This manipulation was based on an important assumption of the 2HBT1 effect: team members will have access to unique information and this often imposes an informational advantage on one member for the problem at hand (Stasser and Titus, 1985, 1987). Our study follows this assumption and extends it to dynamic tasks.

Several studies have extended the 2HBT1 effect to tasks within *dynamic* environments using symmetrical teams (e.g., Glynn and Henning, 2000; Shanks et al., 2013; Räder et al., 2014; Tolsgaard et al., 2015; Abbott et al., 2021). For example, Glynn and Henning (2000) observed that two-person teams were more accurate and faster than individuals on a dynamic teleoperation task. During this task, participants were seated at a computer and were instructed to guide an object through a complex path using joysticks.

Different operational conditions (i.e., task characteristics) have been investigated in several studies focusing on dynamic environments. For instance, prior research added events (or “roadblocks”) to tasks that cause a sudden and unfamiliar change in the operational environment which disrupts team functioning and performance. These studies used this approach to examine *processes* relating to teamwork (e.g., team situation awareness) but not performance outcomes. Similarly, across several studies, Cooke and Gorman and colleagues inserted events into Uninhabited Aerial Vehicle (UAV) missions by introducing either a new target, equipment failure, or the appearance of an enemy threat (Gorman et al., 2005, 2006, 2010; Cooke et al., 2009). These events required behavioral adaptation, and greater coordination between team members to maintain/recover performance (Cooke et al., 2009). The study also focused on the *process* rather than performance outcomes. Importantly, the authors described that these events may have a greater impact on team performance than individual performance because they disrupt a team's shared understanding of a task (e.g., team situation awareness and shared mental models) which requires additional communication and coordination to recover performance. For example, when a sudden and unfamiliar event occurs during a dynamic task, a team must communicate to develop an accurate shared understanding of the altered environment and to coordinate team behavior. We will extend the key results of these studies, using dynamic task conditions, unexpected events, and focusing on the communication processes as well as the performance outcomes.

Communication is cognitively and temporally demanding (MacMillan et al., 2004). Thus, performance recovery may require more time and greater cognitive resources for teams than individuals. It is this diversion of cognitive resources away from the task that may harm team performance, particularly for naïve teams who have no prior experience working together. Hence, it is essential to determine the performance outcomes of teams, and to compare them with individual outcomes, both under different operational conditions. It may be the case that individuals, on average, have greater performance than naïve teams under specific operational conditions. We expect that when operating conditions change and increase the cognitive demands of the task, the communication processes in naïve teams may deteriorate their performance, possibly reversing the 2HBT1 effect.

In the present study, participants completed a dynamic driving simulation under two operational conditions (normal and fog) as either individuals or asymmetrical, distributed teams. Teams were composed of a driver and a navigator. The driver's role was to navigate a vehicle as quickly and safely as possible to the target destination that was directed by arrows. The navigator's role was to observe the driver's environment and provide information and instruction that would help the driver achieve the goals. The fog condition was an unexpected event that disrupted the normal condition and increased the cognitive workload of the driver, which disrupted performance and required adaptation to recover. During the normal condition, both team members were exposed to the same conditions and had access to similar information, however, during the fog condition, team members were exposed to different conditions and information which conferred an informational advantage upon the navigator. Performance was measured using the driver's accuracy and speed (which is a proxy for time). We did not expect the 2HBT1 effect to emerge under both operational conditions for both performance metrics. Given that we have limited cognitive capacity, these effects may depend on team communication which is cognitively and temporally demanding, especially in naïve, asymmetrical, and distributed teams.

### Team communication

Communication is an essential process when working in a team (Keyton et al., 2010) and has been shown to distinguish high and low performing teams (Cooke et al., 2007). Broadly, team communication is the exchange of information between team members (Adams, 2007). Thus, teams have been conceptualized as information processing units (Hinsz et al., 1997; Tindale and Kameda, 2000). Team communication plays a central role in models of team performance. Theory posits that it is vital for most team processes (e.g., coordination) and emergent states (e.g., shared mental models and team cognition) and has a direct and indirect relationship with team performance (e.g., Kozlowski and Klein, 2000; Marks et al., 2001; Ilgen et al., 2005; Mathieu et al., 2008; Kozlowski and Bell, 2013). Specifically, teams use communication to share information about a task (Salas et al., 2005) and situational factors (MacMillan et al., 2004) to develop a shared understanding (Rouse and Morris, 1986; Endsley, 1988), resolve misunderstandings (Fletcher and Major, 2006), facilitate team coordination, and create strategies (Marks et al., 2001). How best to measure team communication is still a debated question in the team literature.

#### Measurement

Several of the most common metrics of communication are the volume, quality, content, and patterns. Each captures different properties of communication (see below) and all relate to team performance to varying degrees.

*The volume* of communication refers to measures of the duration of speech and frequency of speaking turns (Bunderson and Sutcliffe, 2003; Woolley et al., 2010). This approach is quick and easy to administer, however, it ignores the content of communication and its accuracy. A recent meta-analysis found that measures of communication frequency had weaker associations with team performance than communication quality, which is more difficult to capture using objective metrics rather than self-reports (Marlow et al., 2018). Furthermore, communication is cognitively demanding (MacMillan et al., 2004), so measures of volume which only capture the quantity of communication may be proxies for cognitive load. In this research we account for these caveats by using the volume as a baseline rather than the only metric.

*The quality of communication* refers to the clarity, accuracy, and timeliness of information shared between team members. Higher quality communication tends to be associated with higher team performance (Hirst and Mann, 2004; González-Romá and Hernández, 2014). When it is assessed, it is typically measured using a subjective self-report questionnaire administered after a team task, that asks participants to rate communication quality using a Likert scale (Marlow et al., 2018). This method provides an overall subjective rating of team communication. It is also quick and easy to administer but has several critical limitations. Firstly, it does not consider the content of communication or its objective accuracy. Second, self-report measures are susceptible to numerous biases, such as recall and social desirability, which may distort responses (e.g., Sackett, 1979; Arnold and Feldman, 1981). Lastly, this approach treats communication as a static process despite research demonstrating it is dynamic and dependent upon the situational characteristics of the operational environment (Cooke et al., 2009). According to Smith-Jentsch (2009), there is a dearth of research that has assessed the accuracy of knowledge quality shared between team members using more objective measures. We will attempt to resolve this using a novel method, based on manual content analysis, that we developed for this study.

*Manual content analysis* is a common approach to analyze the content of communication, and an alternative or supplement to self-reports. It requires a researcher to select or develop a classification scheme to reduce the complexity of communication data to several categories that represent both the linguistic content of a team's interactions and cognitive processes (e.g., knowledge sharing, information processing, and planning). For example, Bowers et al. (1998) coded all speaking turns into one of seven categories which described the content of each speaking turn by team members. These categories were: (1) uncertainty, (2) action, (3) acknowledgment, (4) responses, (5) planning, (6) factual information, and (7) non-task related statements. They reported that lower performing teams had a higher rate of non-task related communication, and higher performing teams acknowledged or responded to verbal acts at higher rates than lower performing teams. The major limitation of implementing this approach is the large cost in time and resources, and a lack of cross-validation in classifying behaviors into different categories.

We will apply this approach, however, in a novel way to quantify the quality of communication within each team. We define the quality of communication as speech that is both, (1) accurate for the present situation and (2) delivered at an appropriate time which allowed the other team member to respond while the information/instruction was valid. Assessing the content of communication has the additional advantage of allowing for an assessment of communication patterns.

*Communication patterns* are recurring sets of verbal behaviors that develop when people spend time working together (e.g., Bowers et al., 1998; Fischer et al., 2007; Gorman et al., 2012). For example, Stachowski et al. (2009) observed that higher performing teams engaged in fewer, shorter, and less complex patterns of communication than lower performing teams. Typically, however, the identified patterns ignore the semantic content of communication, missing out on the import aspects of the quality (Wildman et al., 2014). We will also apply this approach in a novel way to identify stable and recurring sets of speech that represent patterns of *quality* of communication in each of the operational conditions during our dynamic simulation.

#### The cost of communication

The 2HBT1 effect indicates that, on average, teams tend to perform better than individuals across a range of tasks under the same and different conditions, however, not all teams outperform individuals. This may be, at least in part, because teams have limited cognitive resources and communication is cognitively and temporally demanding; thus, efficient communication is vital for team performance (MacMillan et al., 2004). MacMillan and colleagues observed that the more speech required to communicate a piece of information the greater the cognitive overhead (i.e., time to communicate the information and cognitive resources required to process the information). Depending on the cognitive resources available to team members, the performance of some teams may thrive, while others may succumb with higher levels of communication. This is consistent with the literature on information overload which suggests that a higher frequency of communication is likely to contain more irrelevant and distracting information (see Edmunds and Morris, 2000 for review). Furthermore, it is consistent with the literature on mobile phone use while operating a motor vehicle. This research reveals that drivers who communicate while driving tend to take longer to detect and respond to changes in their environment (e.g., McKnight and McKnight, 1993; Alm and Nilsson, 1994; Lamble et al., 1999; Cooper et al., 2003) and tend to compensate for the increased cognitive workload by driving at lower speeds and more cautiously (Alm and Nilsson, 1994; Haigney et al., 2000; Cooper et al., 2003). Generalizing these findings to our dynamic task embedded within the driving simulation, it appears that communication would have a negative impact on the driver's speed (i.e., time), and possibly accuracy. That is, to maintain accuracy drivers will tend to reduce their speed to compensate for higher levels of communication. There are several implications of these findings for the present study and our novel metrics of the quality of communication.

#### Team communication in the present study

To improve team performance and the efficient allocation of organizational resources, it is important to understand when communication may be helpful and when it may be harmful to team outcomes, especially in dynamic environments that utilize asymmetrical, distributed teams. We expected that teams would have higher accuracy than individuals during the normal condition but no different, or possibly lower accuracy, during the fog condition when demands of the task and the need for communication increase. We also expected that teams would drive slower than individuals during both the normal and fog conditions. The rationale for these predictions is 3-fold: (1) communication is cognitively demanding and people tend to drive slower when engaged in communication; (2) the driver would have a moderate cognitive load during the normal condition, thus, enough available cognitive capacity to attend to communication without diverting resources away from the task of driving and reducing accuracy; and (3) the driver would have a high cognitive load during the fog condition, thus, to attend to communication they would be required to divert cognitive resources away from the task of driving which would reduce accuracy. Within teams, we expected that only the driver's performance would be impacted by communication during the fog event. We did not expect the navigator's performance to be impacted during either condition because of the nature of their role: the cognitive load for the navigator should be lower than the driver's during the normal condition (i.e., they should have cognitive resources to spare) and should not increase substantially during the fog condition when their operating environment appears the same as during the normal condition.

Many studies fail to assess the psychometric properties of the derived communication variables (e.g., internal consistency; Wildman et al., 2014). This is an important step as it allows the appropriateness of communication metrics to be evaluated and for the investigation of stable latent structures of communication. We developed a novel method of measuring the quality of team communication, using multiple iterations of a driving simulation, that permitted these analyses. We then used these novel metrics of communication, together with the traditional metrics of communication volume, to predict team accuracy (collisions) and time (speed). We also compared their magnitude of prediction, controlling for other theory-driven constructs. We expected that our novel measure of communication would be a stronger predictor of accuracy while the volume of communication would be a stronger predictor of speed.

### Simulation-based assessment

In the present study, we employed a high-fidelity driving simulation that we developed in previous research (Kleitman et al., 2022)^1^. Simulations are open-ended, rule-based discovery spaces that allow players to engage with artificial problems that result in quantifiable outcomes (Salen et al., 2004, p. 80). They are well-suited to the assessment of complex skills and behaviors in dynamic environments because, compared with traditional assessment tools, they provide players with a “free-play” environment (Shute and Ke, 2012; Mislevy, 2013); and allow for a better representation of the physical and psychological characteristics of a task (i.e., higher fidelity; Bowers and Jentsch, 2001; Beaubien and Baker, 2004).

### The role of individual differences

Driving is a complex task that requires vision, visual perception, physical control, emotional control, information processing, and executive functions (Anstey et al., 2005; Mathias and Lucas, 2009; Asimakopulos et al., 2012). Individuals differ in these traits and abilities; thus, we measured a range of psychological variables that relate with performance on a driving task and/or team performance. In this research we controlled for these relevant individual differences.

*Executive functions* are a collection of mental processes that are used when one is required to concentrate on a task and pay close attention (Diamond, 2013). There are three core executive functions. Inhibitory control is the ability to resist temptation and avoid acting impulsively or prematurely (De Jong et al., 1995). Working memory is the ability to hold information in one's mind and perform mental work with it (Baddeley, 1992). It allows one to identify connections between ostensibly unrelated things. Cognitive flexibility is the ability adapt to new task demands or rules and, if they are failing, the ability to change one's approach to solve a problem (Davidson et al., 2006). Each of these functions are essential during dynamic tasks, like our simulation, where the operational environment may change at any moment.

*Cognitive ability* refers to one's capacity to process information, and to plan and execute courses of action to achieve one's goals (Carroll, 1993). Two major types of cognitive abilities are fluid intelligence and crystallized intelligence (Cattell, 1971, 1987). Fluid intelligence refers to reasoning ability, and the ability to generate, transform, and manipulate different types of information in real-time to achieve one's goals. Crystallized intelligence refers to knowledge that is acquired through experience, culture, and prior learning. Prior research has shown that across a range of tasks higher cognitive ability is associated with higher team performance (Devine and Philips, 2001; Bell and Kozlowski, 2002). We focused on Fluid Intelligence as it appears more relevant than Crystallized Intelligence for our dynamic driving simulation.

*Personality*: an abundance of research has examined the relationships between team performance and personality, particularly the Big Five personality traits. Meta-analyses have found that Agreeableness, Conscientiousnes, and Openness to Experience had significant positive relationships with team performance (Bell and Kozlowski, 2002; Peeters et al., 2006). Furthermore, drivers who are lower on Conscientiousness are more likely to be involved in driving accidents (Arthur and Graziano, 1996; Sümer et al., 2005). Schneider (2004) observed that people higher on Neuroticism tend to have poorer task performance when under stress. Dynamic simulations, such as ours, can be stressful experiences because the environment and demands of the task may rapidly change, thus, Neuroticism, which indicates one's emotional stability, may also be relevant for the present study.

*Demographic characteristics:* The proportion of females in a team has also been shown to relate with team performance across a range of static tasks. Woolley et al. (2010) observed that teams with a higher proportion of females tended to perform better than teams with a lower proportion of females.

We controlled for these individual differences variables when predicting team performance during our driving simulation. Given their direct relevance to a dynamic simulation, we also controlled for driving experience and gaming experience.

### The present study

Participants in our study completed a dynamic driving simulation under two sets of conditions, each with two levels: grouping (individual vs. team) and operational condition (normal vs. fog). Grouping was a between-subjects condition where participants completed the driving simulation as either individual drivers or distributed two-person teams which consisted of a driver and a navigator that operated a UAV. The operational condition was a within-subjects condition where participants were exposed to a normal and a fog environment during each lap of the driving simulation. Normal conditions were characterized by high visibility for both the driver and navigator, thus, both team members had access to similar information about the environment from their differing points of view. Whereas, fog conditions were characterized by a sudden onset of dense fog that greatly reduced the driver's but not the navigator's visibility. The navigator's view of the driver's environment was unaffected by the fog as the navigator had access to additional information from the UAV which was presented in a separate window (described in more detail in the Method). Thus, team members were exposed to different information about the environment and the navigator had an informational advantage. All other environmental characteristics were consistent across normal and fog conditions. We recorded each team's communication during the simulation. They were coded as described and related to two metrics of the driver's performance: accuracy (collisions) and time (speed). The different operational conditions allowed us to examine whether the 2HBT1 effect and the relationship between communication and performance depended on the characteristics of a task which may introduce an informational advantage. The simulation is described in further detail in the Methods section (see also Kleitman et al., 2022).

As noted earlier, we quantified the quality of knowledge communicated between team members (i.e., accuracy and the appropriateness of its timing) by developing a novel coding system and manually coding each speaking turn into different behavior categories that captured team cognition. The five behavior categories were (1) observation, (2) command instruction, (3) inquiry, (4) redundant, and (5) frustration. For observation and command instruction we further indicated whether the communication was helpful (high quality: accurate and well-timed) or harmful (low quality: inaccurate and/or ill-timed). For each team, we then calculated the frequency of each communication category during each of the operational conditions for each lap. See the Methods section for further detail about the novel communication coding system.

Once these metrics were obtained a factor analysis was utilized to identify stable patterns of communication during the two operational conditions. While it was difficult to formulate specific hypotheses about the factor structure of the communication metrics a priori, given the novelty of this approach, we can still make exploratory predictions. Our communication coding targeted helpful (high quality: accurate and well-timed) and harmful (low quality: inaccurate and ill-timed) communication. Thus, we expected these two respective factors capturing helpful and harmful communication patterns to emerge. We then examined the relationship between the extracted communication factors and team performance separately for the two operational conditions using hierarchical multiple regression models.

The overarching goal of the present study was to better understand distributed and asymmetrical team performance during a dynamic task with changing operational conditions. To investigate this goal, we used a mixed design (grouping: between-subjects; operational condition: within-subjects). Accordingly, the aims and relevant hypotheses of the present study were:

AIM 1: To investigate whether the 2HBT1 effect emerged during different operational conditions on a dynamic task.

We expected that teams would be more accurate (fewer collisions) than individuals during the normal condition but not during the fog condition given the cognitive overhead associated with additional communication. Similarly, we expected that teams would drive slower than individuals in the normal and fog conditions. Thus:

*Hypothesis 1a-b: Teams would have fewer collisions (a) and drive slower (b) than individuals in the normal condition*.
*Hypothesis 1c-d: There would be no difference on collisions (c) but teams would drive slower (d) than individuals during the fog condition*.

AIM 2: To examine the relationship between the patterns of communication quality and team performance during the two operational conditions (normal and fog), after controlling for theoretically important individual differences variables. We expected that a hypothesized “helpful” communication factor would negatively predict collisions and a “harmful” communication factor would positively predict collisions in both operational conditions.

We also expected that, given the cognitive overhead associated with communication (MacMillan et al., 2004) and that drivers tend to go slower when communicating (Haigney et al., 2000), both communication factors would negatively predict speed in both operational conditions. That is, regardless of the quality of communication, higher levels of communication would be detrimental to speed.

*Hypothesis 2a-b: Helpful communication would negatively predict collisions (a) and speed (b) in the normal and fog conditions*.
*Hypothesis 2c-d: Harmful communication would positively predict collisions (c) and negatively predict speed (d) in the normal and fog conditions*.

Aim 3: To investigate whether our novel metrics of communication quality would be stronger predictors of team performance than traditional measures of the volume of communication (i.e., duration of speech and number of speaking turns). That is, given that the content of communication should be useful for avoiding collisions, we expected that our metrics of communication quality would be stronger predictors of accuracy than the volume of communication that ignores the content. In contrast, given that communication comes with a cognitive overhead and drivers tend to go slower when communicating we expected that our metrics of communication would be weaker predictors of speed compared with the volume of communication. Thus,

*Hypothesis 3a-b: Our novel metrics of communication would be stronger predictors of collisions (a) and weaker predictors of speed (b) than the volume of communication*.

### Statistical analyses

To identify stable patterns of communication quality an exploratory factor analysis (EFA) was conducted on the communication behaviors coded using our novel coding system. To examine hypotheses 1a-d we conducted a series of two-way mixed design ANOVAs to investigate the differences between grouping (between subjects: individuals vs. teams) and operational conditions (within-subjects: normal vs. fog) on accuracy (collisions) and speed. To examine hypotheses 2a-d, a series of hierarchical regression analyses were conducted to investigate the relationship between the extracted communication factors and team performance (accuracy and speed), after controlling for the “effects” of known common causes. To examine hypotheses 3a-b we reran the same hierarchical regression analyses from hypotheses 2a-d using the volume of communication measures as independent variables instead of the extracted communication factors. We then performed a qualitative comparison of the beta coefficients for the extracted communication factors with those for the volume of communication. All analyses were conducted using R.

## Methods

### Participants

In return for partial course credit, 316 Australian undergraduate psychology students completed the study either alone or as an asymmetrical two-person team (213 females, 103 males, mean age = 19.80, SD = 4.13). A total of 22 participants (12 individuals and five teams) were excluded from analyses (see Supplementary material for details). The final sample included 294 participants (196 females, 98 males, mean age = 19.80, SD = 4.12). One hundred and thirty-four participants completed the driving simulation as individuals (87 females, 47 males, mean age = 19.80, SD = 3.35) and 160 participants completed the driving simulation as 80 two-person teams (109 females, 51 males, mean age = 19.70, SD = 4.69). Although the two samples were unequal, both were deemed appropriate for the analyses planned. That is, about 10 datapoints were present for each variable included in our analyses (Tabachnick et al., 2007), and our sample compared favorably with other studies of teams, which used samples of between 15 and 43 teams (Sniezek and Henry, 1989; Glynn and Henning, 2000; Gorman et al., 2005; Bahrami et al., 2010; Koriat, 2015).

### Measures

#### Raven's advanced progressive matrices

This test is a measure of abstract reasoning (Raven, 1938–65). Each trial presented a 3x3 matrix of abstract figures following a pattern horizontally and/or vertically. The bottom right figure was blank, and participants decided which of eight alternatives completed the pattern. A 20-item version (of 36) was used to save time. Internal consistency estimates are acceptable to good for accuracy (Cronbach's Alpha = 0.68–0.86) and excellent for Confidence (Cronbach's Alpha =0.84–0.96: Cronbach, 1951; Jackson and Kleitman, 2014; Jackson et al., 2016, 2017; Blanchard et al., 2020).

#### Random number-letter switching test

This executive function task is a measure of mental set shifting (i.e., cognitive flexibility; Monsell, 2003). For each trial, an instruction to focus on “letter” or “number” was flashed on the screen, followed by a letter and number displayed together. For example, if the instruction was “letter” and “A6” appeared on the screen then participants determined whether the letter on the screen was a vowel or consonant. If the instruction was “number” then participants determined whether the number on screen was odd or even. If the instruction matched the previous trial, then the trial was referred to as repeat. If the instruction changed then the trial was referred to as switch. The measure contained 16 practice trials followed by 72 test trials. This measure was used to calculate the number of repeat errors, number of switch errors, response time for repeat trials when the response was correct, response time for switch trials when the response was correct, and switch cost (average switch response time when correct minus average repeat response time when correct).

#### Flanker test

This executive function task is a measure of inhibition (Eriksen and Eriksen, 1974). For each trial, a sequence of five horizontally aligned arrows appeared on the screen with the center arrow either pointing left or right. The other four arrows either point in the same direction (congruent trial) or the opposite direction (incongruent trial) as the center arrow. Participants responded as quickly as possible by indicating whether the center arrow pointed left or right. This measure contained 30 practice trials with feedback followed by 100 test trials without feedback. This measure was used to calculate the number of congruent errors, number of incongruent errors, response time for congruent trials when the response was correct, response time for incongruent trials when the response was correct, and inhibitory cost (average incongruent response time when correct minus average congruent response time when correct).

#### Running letter span

This task is a measure of working memory (Pollack et al., 1959; Broadway and Engle, 2010). For each trial, participants were instructed to recall the last *n* letters then they saw a sequence of individually appearing letters which flashed on their screen. They were not told how many letters would be shown in total and had to recall letters in the order they appeared. For example, they were instructed to remember the last 2 letters and the sequence “X Y T R S” appeared then the answer was “R S”. The number of letters to be recalled (*n)* ranged from three to seven and sequences ranged from five to nine letters. The task contained five practice trials with feedback and 15 test trials without feedback. Internal consistency estimates are excellent for accuracy on this test (Cronbach's Alpha =0.85; Broadway and Engle, 2010).

#### Mini international personality item pool

This 20-item questionnaire is a measure of the Big Five personality dimensions: Agreeableness, Conscientiousness, Extraversion, Intellect, and Neuroticism (Donnellan et al., 2006). Participants rated statements, such as “Am the life of the party”, using a five-point Likert scale from (1) *Very inaccurate* to (5) *Very accurate* (Donnellan et al., 2006). There were four statements for each personality dimension. Each dimension has demonstrated acceptable internal consistency (Cronbach's Alpha range = 0.65–0.77; Donnellan et al., 2006).

### Driving simulation

We employed a driving simulation that we developed in previous research (Kleitman et al., 2022). During this simulation, participants completed a simulated emergency driving course in an urban environment as either an individual or an asymmetrical, distributed two-person team. All participants in the “individual” condition performed the role of a driver and participants in the “team” condition performed the role of either driver or navigator. The participants in the “team” condition did not know each other prior to their participation in the study. All participants received the same general instructions about the simulation (described below) relevant to each role.

**Figure 1:**
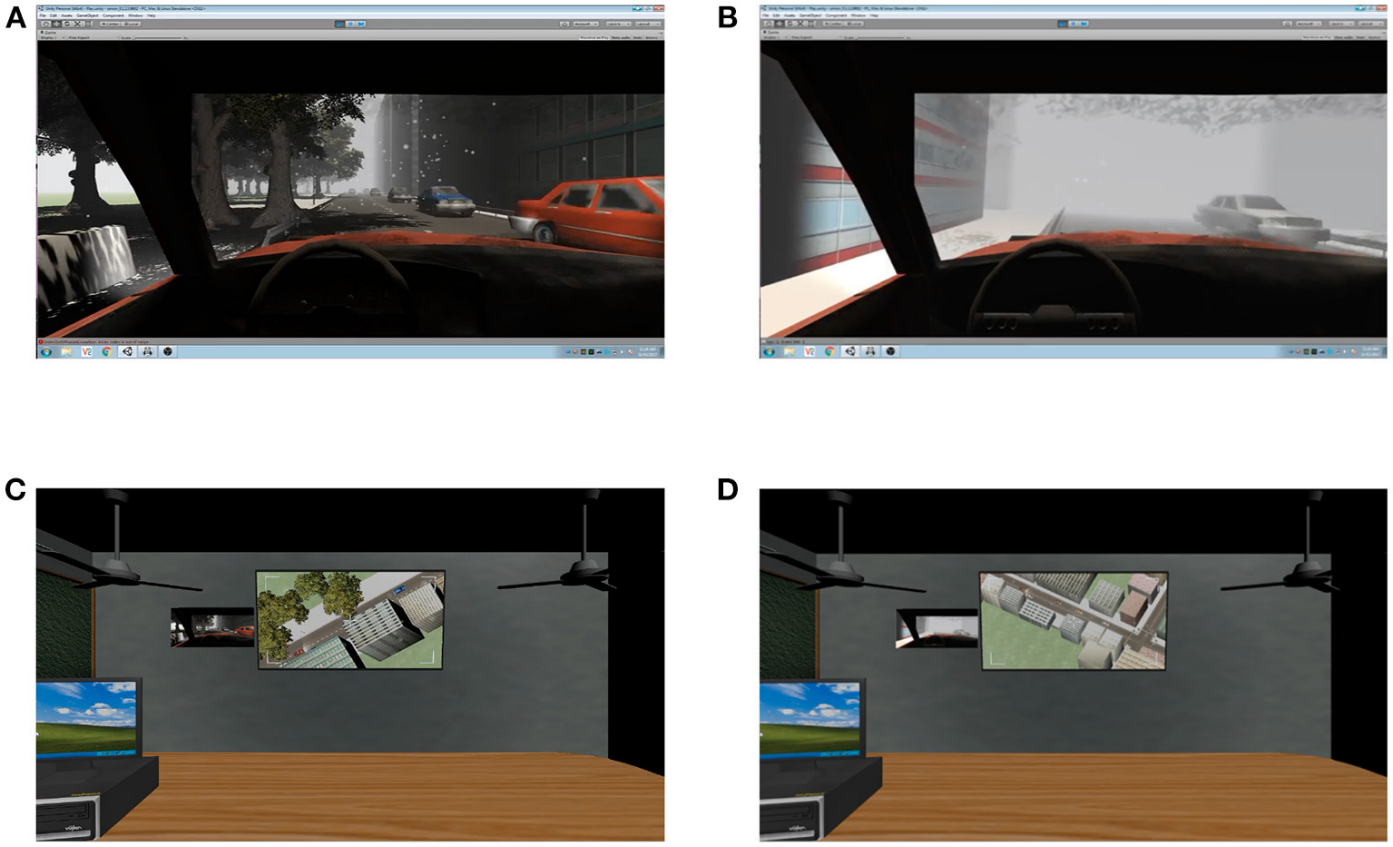
The driver's screen during the normal condition **(A)** and fog condition **(B)** and the navigator's screen during the normal condition **(C)** and fog condition **(D)**.

The goals of the simulation were to deliver essential supplies during unusual weather conditions while staying on a course directed by arrows with or without the assistance of a navigator. The “driver” was permitted to ignore typical road rules (disregarding traffic lights and crossing to the opposite side of the road), but they had to drive as safely and quickly as possible (i.e., minimize collisions and maximize speed). The “navigator” controlled a UAV with a mounted camera that provided a birds-eye view of the driver's environment. In addition to seeing from the UAV's perspective, the navigator's screen also presented the image “transmitted” from the driver's point of view (see Figure 1). Thus, the navigator was presented with the *same* information as the driver (e.g., location and movement of traffic in a driver's vicinity) but also had access to some *unique* information (e.g., the broader landscape). Navigators had to observe the dynamic conditions experienced by drivers and communicate any observations/instructions that would help achieve the team's goals. They could instruct the driver when it was safe to use an oncoming traffic lane or warn them to reduce their speed if they noticed traffic congestion ahead. Participants in the team condition were seated in different rooms (consistent with the scenario) so they could not see each other but they could freely communicate via headsets using a push-to-talk intercom system. This allowed us to record the speaker and receiver's identities, and the duration, timing, and content of speech.

Unbeknown to participants, the simulation was divided into five unique laps that started and ended at the same location, and a single “fog” condition occurred during each lap. All other times were classified as “normal” driving conditions. The normal and fog conditions were designed to last approximately the same duration of time. Five laps allowed us to assess the psychometric properties of the communication measures and measures of performance in the simulation. Figure 1 presents the computer screen visible to the driver and navigator during the normal and fog conditions.

#### Variable computation

Task performance was evaluated based on the driver's collisions and speed (outcome variables). Collisions were defined as events in which the driver's vehicle came into contact with any other vehicle or object in the environment (moving or stationary); speed was defined in notional kilometers per hour. The frequency of each communication type was recorded and the method is described in the next section. These variables were computed for each lap, during the “normal” and “fog” conditions.

### Quantifying team communication

#### Categories of communication behavior

To assess team communication, we used a common approach (e.g., Predmore, 1991; Bowers et al., 1998; Krippendorff, 2004) where each speaking turn was assigned to one of several role contingent categories. To define the categories, we first identified unique communication behaviors that captured components of team cognition (Salas et al., 2005). Specifically, we targeted speech that indicated knowledge updating, information processing, and reduced cognitive resources (see Table 1 for examples).

**Table 1:** Information about the communication categories used to code team discussion during the driving simulation.

**Category of communication**	**Component of team cognition**	**Definition**	**Example**
**Navigator**			
Helpful observation	Team knowledge building: a transference of *accurate* individual knowledge to team knowledge.	The information is appropriate and accurate for the driver's current situation.	“There's a lot of traffic up ahead.”
			“There's a right turn coming up in 10 seconds.”
Harmful observation	Team knowledge building: a transference of *inaccurate* individual knowledge to team knowledge.	The information is not appropriate and/or inaccurate for the driver's current situation.	“There's no traffic up ahead.” [When the road ahead has numerous vehicles.]
			There's a right turn coming up ahead.” [When the arrows at the upcoming intersection are pointing left.]
Helpful command instruction	Performance monitoring: the navigator *accurately* advises the driver about a future course of action.	The instruction is appropriate and accurate for the driver's current situation.	“Switch lanes to overtake the car in front.”
			“Wait for the traffic light to turn green because there are cars in front and behind your vehicle.”
Harmful command instruction	Performance monitoring: the navigator inaccurately advises the driver about a future course of action.	The instruction is not appropriate and/or inaccurate for the driver's current situation.	“Switch lanes to overtake the car in front.” [When the other lane contains incoming traffic.]
			“Reverse then switch to the other side of the road to avoid the traffic at the red light.” [When the driver's car is stationary at a red light and surrounded by traffic ahead and behind.]
Inquiry	Team knowledge processing.	Seeking new information or clarification of existing knowledge about the task.	“What do you see ahead of you?”
			“Why have you stopped your car?”
Redundant	Reduced cognitive resources (e.g., Increased communication overhead).	The information or instruction is irrelevant for the driver's current situation or task.	“Move into the other lane.” [after the driver has already changed lanes.]
			“I am hungry.”
**Driver**			
Command instruction/observation	Team knowledge building.	The information or instruction relates to their current situation or the task.	“There's a red light ahead of me.” [When asked why they stopped their car.]
			“I'm currently driving through fog so my visibility is low.”
Inquiry	Team knowledge processing.	Seeking new information or clarification of existing knowledge about the task.	“Can you tell me when there are a lot of cars ahead?”
			Can you tell me what is around me now?” [When they have lost visibility during the fog event.]
Frustration	Reduced cognitive resources.	Driver produces an audible expression of frustration.	“Oh no!” [As the driver's car collides into another vehicle.]
			An audible sigh when the driver is stationary and stuck in dense traffic.

Effectively performing one's role required different types of communication behavior for drivers and navigators. A driver's role was to operate the vehicle and update team knowledge via communication. A navigators's role was to communicate with the driver by updating team knowledge, processing information, instructing, and monitoring the driver's performance. Given that a driver's speech did not impact their performance and that driver's relied on the navigator's communication to guide their decision making and driving behavior, we focused the quality of communication on the navigator's speech.

For the navigator, there were four broad categories of communication behaviors: observation, command instruction, inquiry, and redundant (see Table 1 for definitions and examples). We then assessed the quality of each observation and command instruction by categorizing them as either helpful or harmful. Helpful indicated that the observation or command instruction was *accurate* for the driver's situation and carried the potential to assist the driver's performance. Harmful differed in that it was *inaccurate* for the driver's situation and/or carried the potential to impair the driver's performance.

For the driver, we coded three categories of communication behavior: command instruction/observation, inquiry, and frustration (see Table 1 for examples). We decided to combine command instruction and observation into a single category after a preliminary analysis of the driving simulation recordings. These instances appeared to be of the same nature and the content of a driver's speech did not appear to impact their own performance so we did not assess the quality of these speaking turns. We measured frustration as a proxy for stress (Zheng et al., 2012). Stress reduces the cognitive resources available for a task (Cohen and Cohen, 1980), the amount of communication between team members (Gladstein and Reilly, 1985; Driskell and Johnston, 1998), and team performance (Driskell et al., 1999; Ellis, 2006).

**Figure 2:**
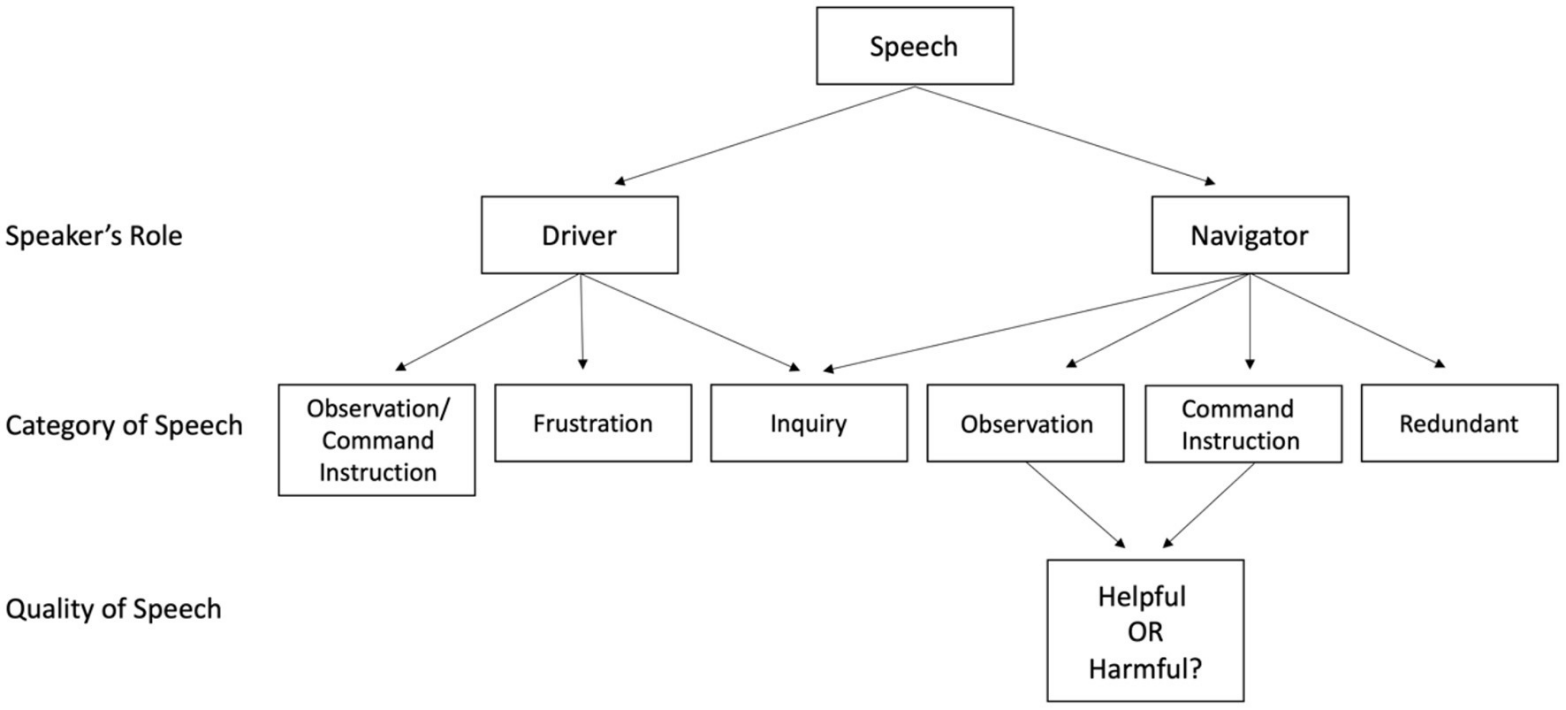
The process of coding each speaking turn during the driving simulation.

After the communication categories were established, independent raters coded all speaking turns during the driving simulation. See Figure 2 for a visual representation of the process of coding each speaking turn. This was facilitated by browser-based coding software developed for this study using JavaScript, HTML, and CSS. Before coding any communication, the raters were trained in the definitions of the coding system and to use the coding software.

#### Coding communication

**Figure 3:**
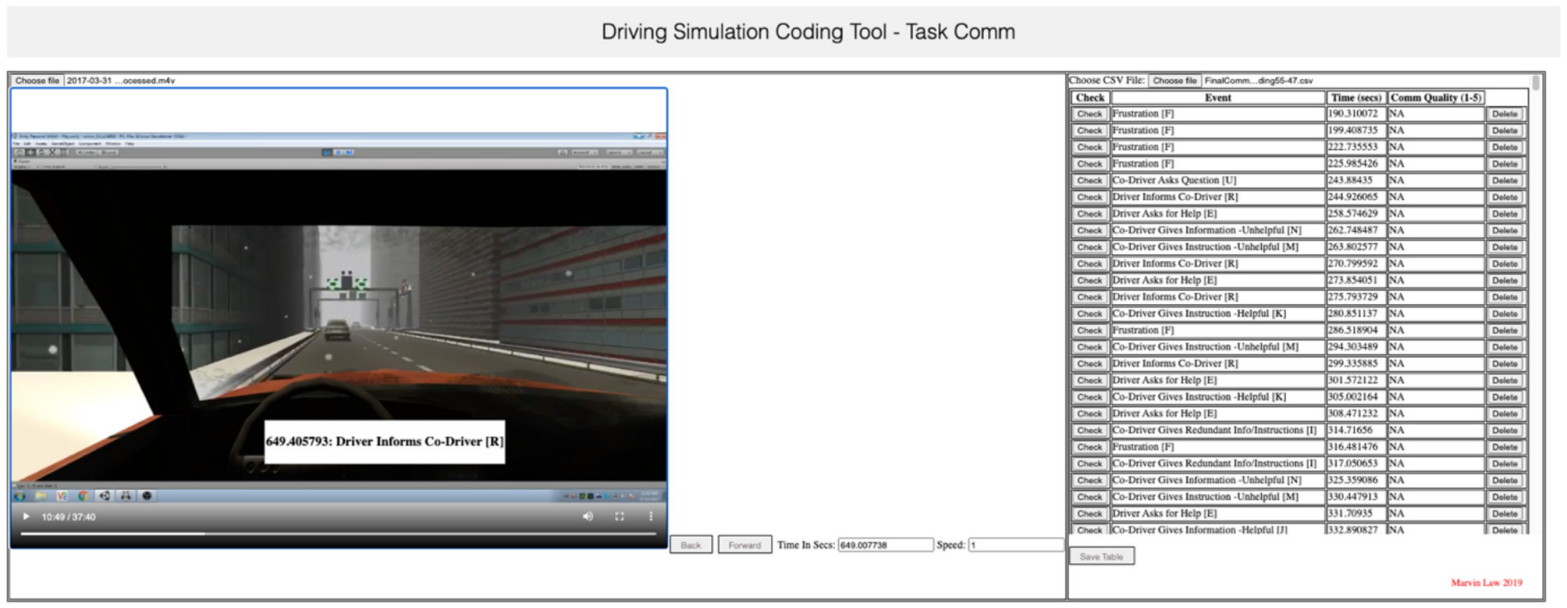
An application developed to code communication during the driving simulation.

The video recordings of drivers' computer screens, which included team discussion, were coded by four independent raters who were naïve to the aims of this study. They coded the videos using the coding application we developed (see Figure 3). It was developed to foster consistency in the coding of communication while watching a recording instead of recollecting it. It enabled the raters to code each speaking turn using a keyboard as they watched a recording on a computer screen. The raters were able to pause, rewind, or fast-forward each recording as needed to accurately code each speaking turn. Timestamps were recorded to allow communication behavior to be mapped onto laps and operational conditions.

Before commencing, all four raters attended an extensive training session which consisted of defining each category of communication, providing examples of each category, demonstrating how to use the coding software, and coding a segment of a recording together as a group. To examine interrater reliability, the four raters then independently coded the *same* recording which was 37 min long and contained 539 speaking turns. Fleiss's (1971) Kappa revealed high interrater reliability overall (0.83). When discrepancies occurred, the raters were instructed on how to code the communication according to the definitions. The remaining recordings were then divided between the raters and coded independently. When coding was completed, an independent review was conducted to improve consistency between raters. Each recording was reviewed in its entirety to check that the coded communication matched the definition of each communication category. If the reviewer disagreed with the category assigned, it was modified to the most appropriate category as indicated by its definition (see Table 1).

### Procedure

Up to four participants completed the 2-h study at a time. All participants were randomly assigned to either the individual or team condition and completed the protocol in the same order. First, background questionnaires assessing demographic information including age, sex, experience with driving, gaming and simulations, and their susceptibility to motion sickness. Then participants completed the driving simulation, followed by the psychometric measures in the order they are described. The background questionnaires and psychometric measures were completed individually. The driving simulation was completed by individuals or two-person teams based on the assigned condition. Ethics approval was granted by the Australian Defence Science and Technology Group's Low Risk Ethics Panel (Protocol Number LD14-16).^2^

## Results

Prior to investigating our aims and hypotheses, we examined descriptive statistics and internal consistency (captured by Omega coefficient) for the simulation derived measures of team performance, individual differences, and the communication measures. This allowed us to check that scores on each variable were as expected, were reliable, and that individuals and teams were similar on the control measures. Omega total (McDonald, 1999) was used to measure internal consistency because we assumed unidimensionality but not tau-equivalence for collisions and speed during the driving simulation. Next, we identified communication patterns using exploratory factor analysis to extract latent components of communication behaviors. Then we examined the aims and hypotheses in the order they are listed.

### Descriptive statistics

#### Simulation derived performance metrics

**Figure 4:**
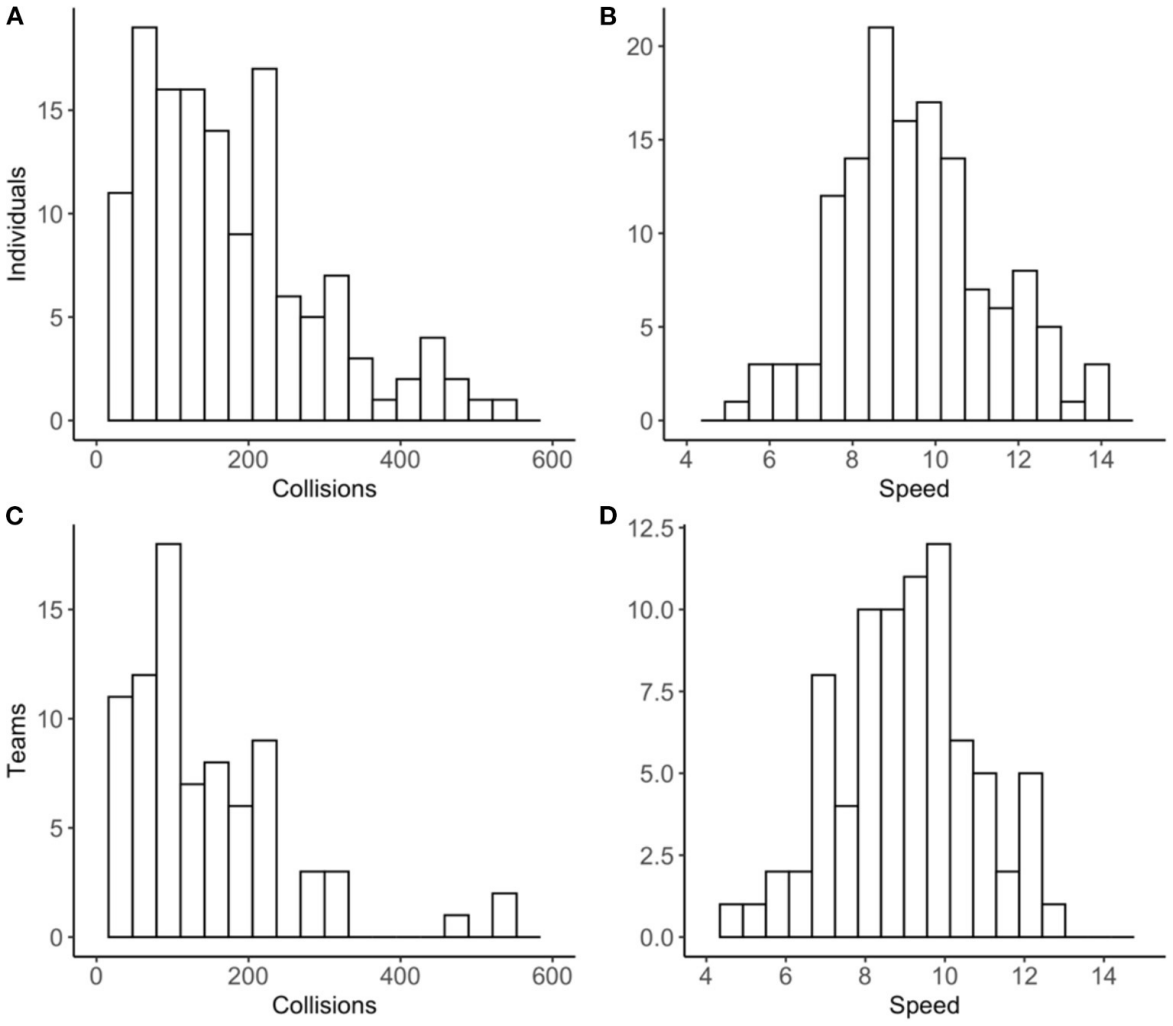
Frequency distributions for individual collisions **(A)** and speed **(B)** overall and team collisions **(C)** and speed **(D)** overall.

Frequency distributions for collisions overall and speed overall (across all laps and operational conditions) for individuals and teams are displayed in Figure 4.

Collisions overall were positively skewed and speed overall was normally distributed for both individuals and teams. The same distribution shapes were observed for each performance metric during normal and fog conditions (see Supplementary material).

Table 2 reports the descriptive statistics and reliability estimates (Omega total) for collisions and speed for individuals and teams overall and during the two operational conditions.

**Table 2:** Descriptive statistics and Omega reliability coefficients for the performance measures (*N* = 80).

		**Individuals**			**Teams**		
		ω_t_	**Mean**	**SD**	ω_t_	**Mean**	**SD**
Collisions	Normal	0.81	100.29	73.12	0.88	76.79	69.18
	Fog	0.82	76.65	50.99	0.73	67.20	46.16
Speed	Normal	0.86	9.60	1.85	0.88	9.20	1.78
	Fog	0.82	9.45	1.87	0.83	8.79	1.78

ω_t_, Omega total.

Reliability estimates ranged from good (0.73) to excellent (0.88) for collisions and speed across the two operational conditions and the two grouping conditions. These values indicate that the duration of our normal and fog conditions were appropriate to capture reliable behaviors involved in the 2HBT1 effect. In this study, we used speed as a behavioral measure of time taken for task completion. This was justified given the strong relationship between speed and time during the normal condition (*r* = −0.69, *t* = −13.90) and fog condition (*r* = −0.60, *t* = −10.40) for all drivers.

#### Communication measures

The descriptive statistics and reliability estimates for the coded communication behaviors are presented in Table 3 and the volume of communication variables are provided in Table 4. These variables were calculated on a subset of teams (*N* = 53). We could not compute them for 27 teams because at least one member's recording of communication was inaudible or missing. All subsequent analyses that include the communication variables are performed on this subset of teams. It is important to note, that there were no significant differences between the full sample of teams and the subset of teams on the simulation derived metrics of performance or the individual differences measures. At the time when the coding was completed, and the software failure was identified, no more data collection was possible. Still, the remaining sample size is consistent with the existing literature on teaming which range from 15 to 43 teams (Sniezek and Henry, 1989; Glynn and Henning, 2000; Gorman et al., 2005, 2006; Bahrami et al., 2010; Koriat, 2015).

**Table 3:** Descriptive statistics for the coded communication behaviors for a unit of distance traveled (*N* = 53).

	**Normal**			**Fog**			***t***
	ω_t_	**Mean**	**SD**	ω_t_	**Mean**	**SD**	
**Driver**							
Observation or command instruction	0.90	0.0025	0.0020	0.81	0.0034	0.0025	−5.13^***^
Inquiry	0.80	0.0019	0.0013	0.83	0.0024	0.0015	−4.43^***^
Frustration	0.89	0.0013	0.0014	0.90	0.0016	0.0017	−2.91^**^
**Navigator**					0.0048	0.0025	
Helpful observation	0.86	0.0041	0.0023	0.81	0.0003	0.0003	−3.39^**^
Harmful observation	0.71	0.0002	0.0003	0.54	0.0055	0.0040	−2.14^*^
Helpful command instruction	0.90	0.0048	0.0032	0.90	0.0006	0.0006	−2.63^*^
Harmful command instruction	0.43	0.0004	0.0004	0.78	0.0010	0.0010	−1.74
Inquiry	0.77	0.0007	0.0007	0.81	0.0010	0.0008	−2.90^**^
Redundant	0.76	0.0010	0.0008	0.75	0.0034	0.0025	−0.25

ω_h_, Omega total.

* *p* < 0.05,

** *p* < 0.01,

*** *p* < 0.001.

**Table 4:** Descriptive statistics for the volume of communication measures for a unit of distance traveled (*N* = 53).

	**Normal**			**Fog**			***t***
	ω_t_	**Mean**	**SD**	ω_t_	**Mean**	**SD**	
**Duration (seconds)**							
Team	0.85	0.131	0.096	0.70	0.078	0.054	6.95^***^
Driver	0.87	0.053	0.056	0.79	0.031	0.027	4.59^***^
Navigator	0.73	0.078	0.058	0.64	0.048	0.033	5.37^***^
**Talking turns**							
Team	0.87	0.016	0.008	0.85	0.019	0.009	−5.20^***^
Driver	0.86	0.004	0.003	0.80	0.006	0.003	−5.52^***^
Navigator	0.87	0.011	0.005	0.87	0.013	0.006	−4.01^***^

ω_t_, Omega total.

*** *p* < 0.001.

Given that, by design, the distance traveled during the normal (Mean = 7,113, SD = 1,397) and fog conditions (Mean = 4,992, SD = 1,080) significantly differed [*t*_(213)_ = 30.75, *p* < 0.001] and that a greater distance traveled provided more opportunities for communication, we computed a ratio for the coded communication behaviors and the volume of communication measures. The ratio represented the average amount of each type of communication for each unit of distance traveled during the two operational conditions. Thus, the descriptive statistics and *t*-tests presented in Tables 3, 4 were computed using these ratio variables. It should be noted that, on average, the driving simulation took 27.76 min (SD = 7.29) to complete and approximately the same amount of time was spent in the normal (Mean = 14.33 min, SD = 3.63) and fog conditions (Mean = 12.44 min, SD = 4.06).

As expected, higher levels of each of the coded communication behaviors occurred during fog conditions compared with normal conditions. There were two exceptions, harmful command instruction and redundant did not differ between the two operational conditions. Reliability estimates were acceptable (>0.60) for all coded communication behaviors except harmful observation during fog conditions (ω_t_ = 0.54) and harmful command instruction during normal conditions (ω_t_ = *0.4*3).

For the volume of communication measures, there were two consistent patterns: (1) there was a greater duration of speech in the normal compared with the fog conditions; and (2) a greater number of talking turns in the fog compared with normal condition. Reliability estimates were acceptable (ω_t_ > 0.60) for all volume of communication variables.

#### Individual differences measures

Descriptive statistics and measures of internal consistency are presented in the Supplementary material. This is to maintain focus on the performance metrics and communication variables as these individual differences measures are treated as control variables in this study. The means and standard deviations were consistent with previous research using Australian undergraduate students (Jackson et al., 2016, 2017; Blanchard et al., 2020). All reliability estimates were acceptable for research purposes except repeat errors (team ω_t_ = 0.57 and driver ω_t_ = 0.29), switch errors (all levels, ω_t_ = 0.28–0.54), congruent errors (all levels, ω_t_ = 0.39–0.54), and incongruent errors (team ω_t_ = 0.55 and driver ω_t_ = 0.44). As these variables demonstrated largely poor internal consistency they were removed from subsequent analyses. These variables related to inhibitory control and cognitive flexibility. We had two different metrics for each of these constructs: errors and response time. The response time measures demonstrated excellent reliability (ranging from ω_t_ = 0.88–0.95), thus, they remained in the study for our analyses. Reliability estimates for the personality measures ranged between ω_t_ = 0.47–0.79 for individuals and ω_t_ = 0.58–0.79 for teams. Some of the reliability estimates for individuals were low, however, we only used the team measures as control variables to examine hypotheses related to aims 2 and 3. Overall, these estimates were consistent with the previous literature using this brief instrument (Jackson et al., 2016, 2017; Blanchard et al., 2020).

### Exploratory factor analysis using communication variables

Next, we extracted latent factors of communication that characterized different patterns of communication behavior during each operational condition, separately. The correlations between the communication variables and a summary of the results of the exploratory factor analyses (EFA) are presented for the normal condition in Table 5 and the fog condition in Table 6.

**Table 5:** Communication variable intercorrelations and EFA results for the normal condition.

**Comm variables**	**Pearson** ***r*** **correlations**								**Factor loadings**		
	**2**	**3**	**4**	**5**	**6**	**7**	**8**	**9**	**1**	**2**	***h** ^2^*
1. Inquiry (navigator)	0.29^*^	0.73^***^	0.36^**^	0.49^***^	0.35^**^	0.01	0.14	0.39^**^	**0.89**	−0.19	0.67
2. Inquiry (driver)		0.41^**^	0.62^***^	0.35^**^	0.26	0.21	0.25	0.10	**0.64**	0.04	0.43
3. Observation or command instruction (driver)			0.53^***^	0.43^**^	0.39^**^	0.12	0.29^*^	0.23	**0.89**	−0.09	0.72
4. Helpful observation (navigator)				0.38^**^	0.16	0.29^*^	0.20	0.14	**0.71**	0.00	0.50
5. Helpful command instruction (navigator)					0.36^**^	0.01	0.33^*^	0.36^**^	**0.72**	0.00	0.52
6. Harmful command instruction (navigator)						0.48^***^	0.29^*^	0.27	0.22	**0.57**	0.49
7. Harmful observation (navigator)							0.50^***^	0.35^*^	−27	**0.99**	0.82
8. Frustration (driver)								0.26	0.00	**0.74**	0.55
9. Redundant (navigator)									0.18	**0.47**	0.33

Comm variables, communication variables; factor loadings > 0.30 are in bold. *H*^2^, communality.

*** *p* < 0.001,

** *p* < 0.01,

* *p* < 0.05.

**Table 6:** Communication variable intercorrelations and EFA results for the fog condition.

**Comm variables**	**Pearson** ***r*** **correlations**								**Factor loadings**		
	**2**	**3**	**4**	**5**	**6**	**7**	**8**	**9**	**1**	**2**	***h^2^***
1. Inquiry (navigator)	0.23	0.64^***^	0.34^*^	0.42^**^	0.45^***^	0.03	0.11	−0.10	**0.95**	–**0.36**	0.73
2. Inquiry (driver)		0.35^*^	0.58^***^	0.33^*^	0.09	0.16	0.29^*^	0.29^*^	**0.32**	**0.47**	0.46
3. Observation or command instruction (driver)			0.47^***^	0.44^***^	0.29^*^	0.19	0.22	0.10	**0.80**	−0.03	0.63
4. Helpful observation (navigator)				0.38^**^	0.13	0.28^*^	0.06	0.04	**0.59**	0.14	0.44
5. Helpful command instruction (navigator)					0.62^***^	0.05	0.27	0.10	**0.76**	0.02	0.58
6. Harmful command instruction (navigator)						0.25	0.28^*^	0.16	**0.59**	0.10	0.40
7. Harmful observation (navigator)							0.2	0.25	−0.04	**0.61**	0.36
8. Frustration (driver)								0.31^*^	0.03	**0.64**	0.42
9. Redundant (navigator)									–**0.31**	**0.88**	0.64

Comm variables, communication variables; factor loadings > 0.30 are in bold. *h*^2^, communality.

*** *p* < 0.001,

** *p* < 0.01,

* *p* < 0.05.

A pattern of small to large positive correlations was evident between most variables in the normal and fog conditions. We conducted a Principal Component Analysis (with promax rotation) on the communication measures during both conditions. We used Principal Component Analysis instead of Principal Axis Factoring because an ultra-Heywood case was detected using factor analysis. Parallel analysis suggested a two-factor solution that explained 55.89% of the common variance for the normal condition and 51.76% of the common variance for the fog condition.

Consistent with the expectations, for the normal condition, all the helpful and inquiring communication variables loaded positively on the first factor and all harmful and redundant communication variables loaded positively on the second factor. These two factors were named Helpful Exchange and Harmful Navigator, respectively, and had a positive, moderate strength correlation with each other (*r* = 0.42, *p* < 0.01).

Similarly, for the fog condition, the pattern of loadings approximated those found for the normal condition, but there were some subtle differences. All the helpful and inquiring communication variables loaded positively on the first factor. Furthermore, the navigator's harmful command instruction loaded positively and redundant communication loaded negatively on the first factor. The redundant communication variable loaded marginally above 0.30. All negative communication variables except harmful command instruction loaded positively on the second factor. In addition, the driver's inquiry loaded positively, and the navigator's inquiry loaded negatively on the second factor. These two factors were named Helpful Exchange and Harmful Navigator, respectively, and had a positive, moderate correlation with each other (*r* = 0.43, *p* < 0.01).

The correlations between the same factors extracted from the normal and fog conditions was high for Helpful Exchange (*r* = 0.83, *p* < 0.001) and for Harmful Navigator (*r* = 0.78, *p* < 0.001). These large correlations suggest that, despite the subtle differences in the loadings, the communication factors were relatively stable across conditions and appeared to represent the same underlying constructs. The extracted factor scores were used in the subsequent analyses of communication instead of the original communication measures.

### Performance during the simulation

#### Individuals vs. teams

We conducted two-way mixed design ANOVAs to test hypotheses 1a-d which were related to aim one. This was to examine the differences between grouping (between subjects: individuals vs. teams) and the two operational conditions (within-subjects: normal vs. fog) on collisions (ratio; see below) and speed. To account for unequal samples (individual vs. teams), we conducted the ANOVAs using Type II Sums of Squares (Langsrud, 2003).

Collisions ratio: By design, the distance traveled during the normal (Mean = 7,113, SD = 1,397) and fog conditions (Mean = 4,992, SD = 1,080) significantly differed [*t*_(213)_ = 30.75, *p* < 0.001] and there was a significant correlation between distance traveled and collisions during the normal condition (*r* = 0.36, *p* < 0.001) and fog condition (*r* = 0.44, *p* < 0.001). To address this, we computed a *ratio* to represent the average number of collisions for each unit of distance traveled during each operational condition. There was no significant relationship between distance traveled and speed during the normal (*r* = −0.06, *p* = 0.36) or fog conditions (*r* = 0.01, *p* = 0.93) so we used the original speed variables in these analyses. Thus, in the subsequent ANOVAs, we used collisions (ratio) and the original speed as the dependent variables.

**Figure 5:**
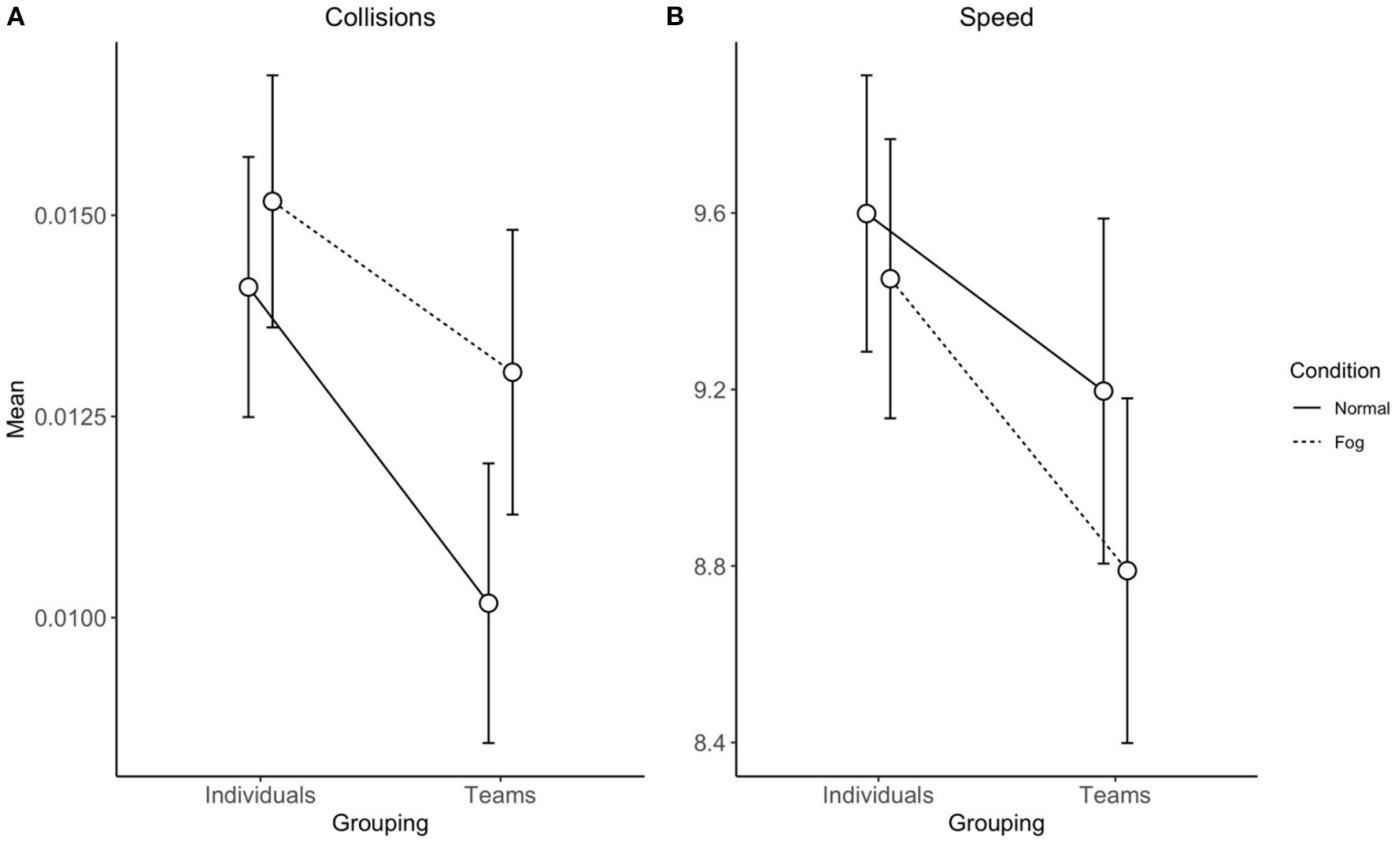
Mean collisions [ratio; **(A)**] and speed **(B)** for individuals and groups during the normal and fog conditions.

Figure 5 displays mean collisions ratio and speed for individuals and groups during the normal and fog conditions. The results of the mixed design AONVA analyses are reported in Table 7.

**Table 7:** Results of two-way mixed design ANOVAs on the differences between grouping (individuals vs. teams) and operational conditions (normal vs. fog) on collisions (ratio) and speed.

	**Group 1**	**Group 2**	**Mean difference**	***F*** **(1,212)**	η^2^
	**Mean (SD)**	**Mean (SD)**			
**Collisions (ratio)**					
Grouping	0.0146 (0.0094)	0.0116 (0.0081)	0.003	6.43^*^	0.027
Operational condition	0.0126 (0.0092)	0.0144 (0.0089)	−0.0017	20.46^***^	0.010
Interaction	–	–	–	5.16^*^	0.002
**Speed**					
Grouping	9.52 (1.86)	8.99 (1.79)	0.53	4.63^*^	0.020
Operational condition	9.45 (1.83)	9.20 (1.86)	0.25	11.11^**^	0.004
Interaction	–	–	–	2.92	–

*** *p* < 0.001,

** *p* < 0.01,

* *p* < 0.05; Grouping, individual vs. team; operational condition = normal vs. fog.

Collisions were significantly lower for teams compared with individuals and significantly greater collisions occurred during the fog compared with the normal condition. The interaction effect was also significant. Supporting hypotheses 1a and 1c, collisions were significantly lower for teams than individuals during the normal condition [*t*_(216)_ = −3.10, *p* < 0.01] and there was no significant difference between individuals and teams during the fog condition [*t*_(216)_ = −1.70, *p* = 0.09]. Supporting hypotheses 1b and 1d, speed was significantly lower for teams than individuals and significantly lower for the fog compared with the normal condition. The interaction effect was not significant.

### Communication as a predictor

Our aim 2 hypotheses were tested using a series of hierarchical regression analyses. To retain adequate power, we reduced the independent variables for teams down to a smaller number of components using EFA. These extracted components were: Executive Function Time which was composed of the response time variables for repeat time, switch time, congruent time, and incongruent time; and Competence which was composed of fluid intelligence, confidence, and working memory accuracy. These EFAs and the correlations between all outcome variables, team composition measures, and control variables are displayed in the Supplementary material.

#### Communication patterns

For the second aim, we examined whether the extracted communication patterns predicted the simulation derived metrics of team performance, after controlling for individual differences variables. The dependent variables were collisions and speed in the two operational conditions (normal and fog). The independent variables were the two extracted communication factors for each operational condition and the following control variables measured at the team level (the average of the driver and navigator): proportion of females, Executive Function Time Factor, Competence Factor, and Neuroticism.^3^ These variables were included as predictors of collisions and speed in separate hierarchical regression models for the normal and fog conditions. The results are shown in Table 8.

**Table 8:** Hierarchical regression analyses for teams during each operational condition with collisions and speed as the criterion (*N* = 53).

**Block**	**Predictor**	**Collisions**								**Speed**							
		**Normal**				**Fog**				**Normal**				**Fog**			
***R***	*R* ^2^	Δ*R*^2^	β	***R***	*R* ^2^	Δ*R*^2^	β	***R***	*R* ^2^	Δ*R*^2^	β	***R***	*R* ^2^	Δ*R*^2^	β
1		0.30	0.09	0.09^*^		0.23	0.05	0.05^†^		0.43	0.19	0.19^**^		0.42	0.17	0.17^**^	
	Proportion females				0.30^*^				0.24^†^				−0.43^**^				−0.42^**^
2		0.33	0.11	0.02		0.38	0.15	0.09		0.52	0.27	0.08		0.46	0.21	0.04	
	EF Time factor				0.07				−0.02				0.21				0.03
	Competence factor				0.02				−0.03				−0.03				−0.03
	Neuroticism				0.10				0.31^*^				0.16				0.18
3		0.52	0.27	0.17^**^		0.46	0.22	0.07		0.57	0.33	0.06		0.59	0.34	0.13^*^	
	Helpful exchange				0.02				−0.03				−0.27^†^				−0.41^**^
	Harmful navigator				0.42^**^				0.29^†^				0.08				0.19

β represents a standardized regression coefficient.

** *p* < 0.01,

* *p* < 0.05,

† *p* < 0.10.

##### Collisions

**Figure 6:**
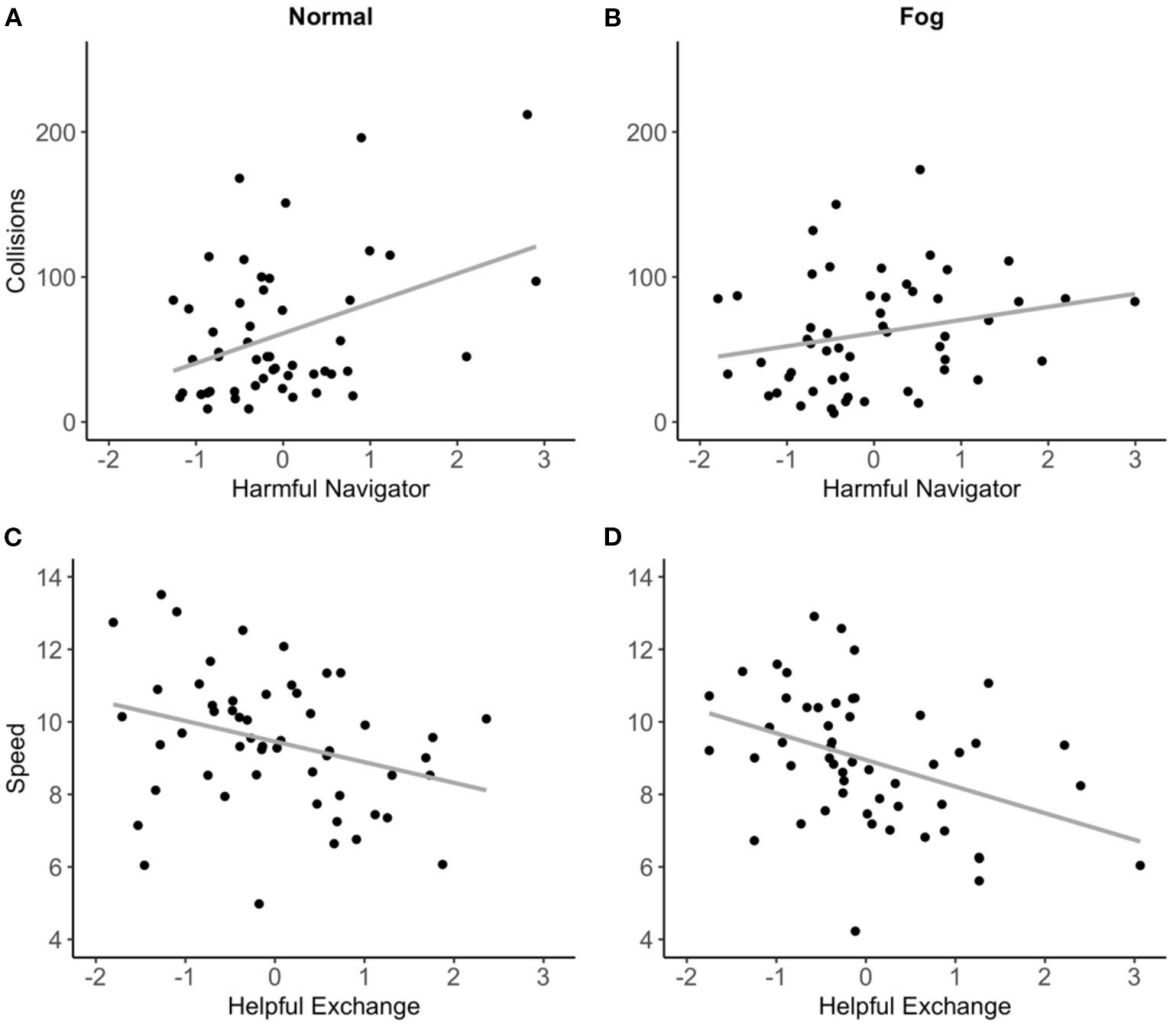
Scatterplots for Harmful Navigator and collisions during the normal condition **(A)**, and collisions during the fog condition **(B)** and Helpful Exchange and speed during the normal condition **(C)**, and speed during the fog condition **(D)**.

In block 1, the proportion of females was a significant predictor of collisions accounting for 9% of its variance during normal conditions (*β* = 0.30; *p* = 0.03). In block 2, the psychological constructs: EF Time Factor, Competence Factor, and Neuroticism were added. None of these variables contributed significantly to the prediction of collisions. In block 3, we added the two communication factors which accounted for a significant amount of additional variance in collisions during the normal condition (Δ*R*^2^ = 0.17, *p* < 0.01) but not the fog condition. In partial support of hypothesis 2c, Harmful Navigator was a significant predictor of collisions during normal conditions (*β* = 0.42; *p* < 0.01) but not the fog condition (*β* = 0.29; *p* = 0.07). Helpful Exchange did not predict either of the performance metrics, thus, hypothesis 2a was not supported. A scatterplot of the scores on collisions during each operational condition and Harmful Navigator is displayed in Figure 6.

##### Speed

Block 1 revealed that the proportion of females significantly accounted for 19% of the variance in speed during the normal condition (*β* = −0.43; *p* < 0.01) and 17% during the fog condition (*β* = −0.42; *p* < 0.01). In block 2, the cognitive factors and personality traits did not significantly predict speed in either of the operational conditions (Δ*R*^2^ ranged from 0.04 to 0.08). In block 3, we added the two communication factors which accounted for a significant amount of additional variance in speed during the fog condition (Δ*R*^2^ = 0.13, *p* < 0.02) but not the normal condition. In partial support of hypothesis 2b, Helpful Exchange was a significant predictor of speed during the fog condition (*β* = −0.41; *p* < 0.01) but not the normal condition (*β* = −0.27; *p* = 0.06). Harmful Navigator was not a significant predictor of speed during the normal or fog conditions, thus, hypothesis 2d was not supported. A scatterplot of the scores on speed during each operational condition and Helpful Exchange is displayed in Figure 6.

##### Volume of communication

Next, to examine hypotheses related to aim 3, we conducted the same hierarchical regression analyses but using the two volume of communication measures as predictors in block 3 instead of the extracted communication factors. The dependent variables were collisions and speed in each of the operational conditions. The purpose of this analysis was to examine whether the communication factors were stronger predictors of collisions but weaker predictors of speed than the volume of communication. A qualitative assessment was performed by comparing the standardized regression coefficients for the different types of communication variables.

The volume of communication was measured using the duration of communication and the number of talking turns for each team. These two variables could not be included together in the regression analyses due to their strong relationship with each other for the normal condition (*r* = 0.72, *p* < 0.001) and the fog condition (*r* = 0.69, *p* < 0.001). Thus, separate hierarchical regression models were conducted with each volume of communication measure as an independent variable in the final block. The results of blocks 1 and 2 were identical to those reported previously in Table 8 so only the results of block 3 are presented in Table 9. Furthermore, we were only interested in the results for the volume of communication variables so the results for the other variables were not reported.

**Table 9:** Final block of results for a series of hierarchical regression analyses for teams during each operational condition using volume of communication measures to predict collisions and speed (*N* = 53).

**Predictor**	**Normal**				**Fog**			
	***R***	*R* ^2^	Δ*R*^2^	β	***R***	*R* ^2^	Δ*R*^2^	β
**Collisions**								
Duration of communication	0.33	0.11	0.00	0.06	0.39	0.15	0.00	0.04
Number of talking turns	0.41	0.17	0.06^†^	0.26^†^	0.40	0.16	0.01	0.13
**Speed**								
Duration of communication	0.63	0.39	0.12^**^	−0.39^**^	0.63	0.40	0.19^***^	−0.47^***^
Number of talking turns	0.58	0.33	0.06^*^	−0.26^*^	0.56	0.32	0.10^*^	−0.33^*^

β represents a standardized regression coefficient.

*** *p* < 0.001,

** *p* < 0.01,

* *p* < 0.05,

† *p* < 0.10.

The regression analyses revealed that neither of the volume of communication variables accounted for a significant amount of variance in collisions, after accounting for the control variables. Furthermore, both volume of communication variables consistently accounted for a significant amount of variance in speed for each of the operational conditions, after accounting for the control variables.

A qualitative comparison of betas for the two types of communication variables supported hypotheses 3a and 3b. The Harmful Navigator factor appears to be a stronger predictor of collisions during the normal condition (*β* = 0.42) and the fog condition (*β* = 0.29) compared with the duration of a communication (*β* = 0.06 and 0.04, respectively) and the number of talking turns (*β* = 0.26 and 0.13, respectively). However, the duration of communication variable accounted for a greater amount of variance and appears to be a stronger predictor of speed during the normal condition (*β* = −0.39) and the fog condition (*β* = −0.47) compared with the Helpful Exchange factor (*β* = −0.27 and −0.41, respectively).

## Discussion

This study was the first to examine the 2HBT1 effect for a dynamic driving simulation with two different operational conditions using naïve, asymmetrical, and distributed teams. We were also the first to investigate the corresponding relationship between two novel measures of communication quality and team performance.

### Two heads are not always better than one

Our results revealed that for naïve, asymmetrical, and distributed teams the 2HBT1 effect depends on the characteristics of a dynamic task and the performance metric (collisions and speed).

*For collisions*, as expected teams performed better (i.e., lower collisions) than individuals during the normal condition but the team advantage disappeared during our brief fog condition. This suggests that teamwork is beneficial under familiar conditions where team members have access to similar information, have a more accurate shared understanding of the task, and there is a lower level of uncertainty (the task's characteristics are stable). When the characteristics of the task suddenly changed (fog condition), two heads performed at the same level as one. This possibly occurred because the fog condition increased the cognitive load on the driver while disrupting a team's shared understanding of the task. It appears, in response to the fog disruption, that drivers relied more heavily on their own understanding of the task and the environment to inform their decisions and behavior. To their detriment, they did not utilize the informational advantage that the scenario bestowed upon the navigator. Thus, during the fog condition, drivers in teams behaved more like individual drivers, and no differences were observed in their performance compared to the individual drivers.

Teams may recover their performance by updating their shared understanding of a task through communication (Gorman et al., 2005; Cooke et al., 2009). To achieve this in our study, either a driver had to notify the navigator of the changed conditions, or the navigator had to notice them. Once aware of the altered environmental conditions, the navigator had to shift their focus to provide more information about the immediate environment surrounding the driver's vehicle. This behavior would increase a driver's accurate knowledge about their environment and potentially allow teams to maintain their advantage over individuals. However, possibly due to the naïve and distributed nature of the teams (e.g., no training) or the brevity of the fog event, this did not occur in our study. Even though navigators had access to more unique information during the fog condition, both drivers and navigators spoke for less time during this condition than the normal condition. Many unexpected events that occur in real-world tasks have a temporary impact, some lasting only minutes like our task (e.g., unexpected weather conditions or equipment malfunction) and others lasting weeks or months (e.g., an earthquake or flood). We suspect that the longer the duration of an altered environmental state the more likely teams are to habituate and recover the 2HBT1 effect. It is important to understand how teams behave and perform under a wide range of conditions that occur in the real world. Our brief fog event may not have been long enough for teams to habituate and recover the 2HBT1 effect. Future research should examine this hypothesis using unexpected events of varying lengths.

*For speed*, as predicted teams performed worse (i.e., slower speed) than individuals during fog conditions but contrary to our hypothesis there was no difference between the two groups during normal conditions. A speed-accuracy trade-off did not account for this result as collisions were lower for teams during the normal condition only and speed was lower for teams during the fog condition only. The lower speed for teams may have been a compensatory strategy to account for a higher cognitive workload during the fog condition compared with individual drivers. That is, drivers in teams experienced approximately the same environmental conditions as individual drivers and had to monitor, process, and respond to a teammate providing them information while controlling a vehicle during the fog condition. Previous research indicates that communication is demanding on cognitive resources (e.g., MacMillan et al., 2004) and when engaged in non-task related communication drivers reduce their speed to compensate for the lower level of attention available for the driving task (e.g., Haigney et al., 2000). In our study, communication was task-related, and teams only reduced their speed relative to individuals during the fog condition when the task's cognitive workload was highest.

Taken together, the results for collisions and speed indicate that the characteristics of dynamic tasks (e.g., normal and event conditions) impact individual and team performance differently. Depending on the performance metric and operational condition, two asymmetrical and distributed heads may be better than one, the same as one, or worse than one. When accuracy is an important performance metric and the operating environment is stable, asymmetrical, distributed teams are preferred to complete dynamic tasks like our driving simulation. However, if the operating environment is known to be volatile, individuals are preferred over these teams, because individual performance approximates team performance and requires fewer resources. If time (speed) is an important metric, then individuals may be preferred for dynamic tasks with stable or volatile operating environments. Either way, the use of asymmetrical and distributed teams on dynamic tasks should be carefully considered as two heads are not always better than one.

### Communication and team performance

Our results revealed that our novel measures of the quality of communication were important predictors of team performance. As expected, we observed that higher levels of Harmful Navigator, which largely consisted of inaccurate and/or mistimed communication, were associated with more collisions during normal conditions. Contrary to our expectations, there was no relationship between Harmful Navigator and collisions during the fog condition or between Helpful Exchange and collisions during either operational condition. During normal conditions, navigators may have provided high-quality observations and command instructions (i.e., Helpful Exchange) that were predominately common knowledge. That is, navigator communication may have contained little unique information, thus, it did not impact driver behavior or have a relationship with accuracy (collisions). This is consistent with the hidden profiles paradigm (Stasser and Titus, 1985) which revealed that teams tend to spend more time discussing common knowledge than unique knowledge. It's also consistent with Mesmer-Magnus and DeChurch (2009) finding that unique information shared between team members has a stronger relationship with team performance than common information. It was surprising that no relationships emerged between the communication factors and collisions during the fog condition when navigators had access to a greater amount of unique information. As theorized for the 2HBT1 results, it appears drivers in teams acted more like individual drivers during the fog condition and did not utilize the informational advantage of the navigator.

As predicted, we also found that higher levels of Helpful Exchange were associated with lower speed during fog conditions. It appears that teams in our study who engaged in higher levels of communication had a greater cognitive workload than teams with lower levels of communication. Thus, they reduced their speed to account for the greater workload. Surprisingly, there was no relationship between Helpful Exchange and speed during normal conditions. This may have occurred because drivers had cognitive resources to spare during the normal condition but not during the fog condition when their visibility was impaired. It appears that Harmful Navigator was not associated with speed in either condition because of the lower frequency with which it occurred. Overall, time (speed) was only associated with high-quality communication when the cognitive load of the task was at its greatest.

### Comparison of communication measures

Next, we conducted a qualitative comparison of the magnitude of the relationship between two performance metrics and two measures of communication (volume and quality). As predicted, the quality of communication was more important for team accuracy than the volume of communication. Furthermore, as hypothesized, the duration of communication was a stronger predictor of time in both conditions compared with Helpful Exchange and Harmful Navigator. This result suggests that the quantity of communication is more important for time than the quality of communication. That is, drivers tend to compensate for the cognitive demands of communication by driving at slower speeds (e.g., Haigney et al., 2000).

Together these findings indicate that the method of quantifying communication should depend on the performance metrics of interest. The content of communication (e.g., communication quality) is more important for accuracy whereas the quantity of communication is more important for time. Furthermore, our study developed a method of quantifying the quality of communication using a more objective measure than self-report questionnaires administered post-task. Our method provides richer and more detailed data that allows it to be mapped to the on-task behaviors captured during a dynamic task. This allowed us to assess both, the relationship between communication and performance and the psychometric properties of communication metrics.

There were subtle differences in the revealed structure of our communication factors during the fog and the normal condition. Some variation in variable loadings was to be expected given that communication behavior can change when the operational conditions change. Nevertheless, very high correlations (above 0.90) confirmed that each of the extracted factors captured the same construct in each condition.

Overall, low-quality communication was negatively associated with accuracy during the normal condition only, while the volume of communication was negatively associated with speed during both conditions. Thus, there may always be some performance cost when naïve, distributed teams whose members have different roles work together on a dynamic task.

### Control variables and team performance

There was no systematic pattern between the theory-driven control variables (personality, intelligence, executive function) and performance metrics. However, one pattern emerged: Teams with a higher proportion of females had more collisions and drove at lower speeds than teams with a lower proportion of females. These findings are consistent with prior research in driving. For example, females tend to have more accidents while maneuvering a vehicle through traffic (Laapotti and Keskinen, 2004) and tend to drive more cautiously (Sarma et al., 2013) compared with males. The finding for accuracy, however, conflicts with recent research on collective intelligence, that suggests teams with a higher proportion of females tend to perform better than those with a lower proportion of females across a broad range of tasks. Although, this research critically differs from their methodology, as they used symmetrical teams and a dynamic task was not a part of their test battery (Woolley et al., 2010).

### Limitations and future directions

Several limitations must be considered. The small sample size prevented us from extracting communication factors for each lap. Communication is a dynamic process; thus, this would allow for an examination of changes in patterns of communication quality over time. The small sample size also precluded us from examining the personality traits as potential mediators or moderators of the relationships between the communication patterns and the performance metrics. Future research should recruit a larger sample to examine these relationships as personality traits may have important indirect relationships with team performance. Our teams were composed of participants who had no prior experience with our driving simulation or working together. The observed relationships between our communication factors and performance metrics may differ for naïve teams composed of task experts, or well-established teams. Furthermore, these relationships may differ for tasks that require a lower cognitive workload. Future research should examine these relationships for tasks with different cognitive workloads. We did not capture any performance metrics for the navigator during the driving simulation, thus, our measures of team performance were computed using the driver's performance only. Future research should quantify the navigator's performance by calculating a range of variables, such as the proportion of time the driver's vehicle was visible to the navigator or the proportion of time the driver's path was visible to the navigator. Participants in our study were provided with information about the task, roles, and their goals but they did not receive task/scenario specific training. In the real world, many teams operating in dynamic environments might receive training. Future research should examine the 2HBT1 effect and our communication results for asymmetrical, distributed teams by first providing training or using task/scenario experts.

Beyond these limitations, future research should replicate and extend our findings by examining: (1) whether teams habituate and recover the 2HBT1 effect under longer event operational conditions; (2) multiple event types to test whether our effects are general or specific to the characteristics of the event; (3) misleading operational conditions to test the two heads are worse than one effect found for accuracy on static tests (see Koriat, 2012, 2015); and (4) extend our results to other types of dynamic tasks.

### Implications

Our results have implications for theory and those tasked with forming naïve and distributed asymmetrical teams to complete dynamic tasks. In our study, the 2HBT1 effect only emerged for accuracy during normal operational conditions which are characterized by stability and low uncertainty. For accuracy during the fog condition and speed during both conditions, teams either did not differ from individuals or performed worse. Thus, under these conditions individuals may be more efficient than teams. There's one exception, when accuracy is an important performance metric and the operational environment is known to be stable (i.e., approximate our normal condition). Those tasked with forming naïve and distributed asymmetrical teams to complete a dynamic task should consider the characteristics of the task and the importance of each performance metric as individuals may be better suited under most conditions.

Our communication results indicated that the content of communication (our novel quality of communication factors) was a more important predictor of accuracy during familiar and stable (normal) conditions but not after a sudden change occurred in the operational environment (fog). The volume of communication, however, was a more important predictor of time (here speed) under both operational conditions. Given the cognitive overhead of communication, training teams to communicate more efficiently may help to maintain the benefit of communication to accuracy and reduce the cost of communication to speed during dynamic driving tasks. Furthermore, our novel measure of communication quality allowed for an assessment of the psychometric properties of communication. This is an important addition to the measurement of team communication in psychological and organizational research and may lead to the identification of stable factors of communication within teams across different conditions and operating environments.

## Conclusion

In conclusion, employing different operational conditions within a dynamic task may provide new insights into the performance of asymmetrical, distributed teams, and the relationship between team performance and important processes such as communication. We demonstrated the conditions under which the performance of asymmetrical, distributed teams thrived and succumbed compared with individuals. We also developed a novel method of measuring the quality of communication that allowed for an assessment of psychometric properties, and allowed for an examination of its relationship with team performance under different operational conditions. Overall, our results suggest that the use of asymmetrical and distributed teams in dynamic environments should be carefully considered, as individuals may be better suited if there are rapid and frequent changes in the operational conditions.

## Data availability statement

The raw data supporting the conclusions of this article will be made available by the authors, without undue reservation.

## Ethics statement

Ethics approval was granted by the Australian Defence Science and Technology Group's Low Risk Ethics Panel (Protocol Number LD14-16). The patients/participants provided their written informed consent to participate in this study.

## Author contributions

MB designed the study, collected and analyzed the data, and prepared the manuscript. SK designed the study, guided MB at each step, and provided feedback on the manuscript and revisions. EA provided feedback on the study design and manuscript, including revisions. All authors contributed to the article and approved the submitted version.

## Footnotes

^1^Kleitman, S., Zhang, L. M., Blanchard, M. D., Law, M. K. H., Xiong, Z., Jackson, S. A., et al. (2020). A ‘Maze Runner' design for the measurement of resilience and adaptability: validation of a novel simulation-based assessment methodology.

^2^The tasks were administered as part of a broader protocol examining a set of hypotheses regarding psychological resilience and adaptability in a high-fidelity simulation. A number of additional measures were captured to examine the hypotheses of interest and are outside of the scope of this paper.

^3^We ran the same analyses including Agreeableness, Conscientiousness, Extraversion, and Openness to Experience. None of these additional personality traits were significant predictors of the performance metrics and the results were largely the same as those reported in Table 8. The one exception was for the normal condition, Helpful Exchange was a significant predictor of Speed. We excluded these personality traits to maintain adequate power in the analyses.

## References

[B1] Abbott E. F. Laack T. A. Licatino L. K. Wood-Wentz C. M. Warner P. A. Torsher L. C.. (2021). Comparison of dyad versus individual simulation-based training on stress, anxiety, cognitive load, and performance: a randomized controlled trial. BMC Med. Educ. 21, 1–10. 10.1186/s12909-021-02786-6 34225722 PMC8256490

[B2] Adams S. K. (2007). Disciplinarily hetero-and homogeneous design team convergence: Communication patterns and perceptions of teamwork (Doctoral dissertation). Virginia Tech, Blacksburg, VA, United States

[B3] Alm H. Nilsson L. (1994). Changes in driver behaviour as a function of handsfree mobile phones-A simulator study. Accid, Anal. Prev. 26, 441–451. 10.1016/0001-4575(94)90035-3 7916852

[B4] Anstey K. J. Wood J. Lord S. Walker J. G. (2005). Cognitive, sensory and physical factors enabling driving safety in older adults. Clin. Psychol. Rev. 25, 45–65. 10.1016/j.cpr.2004.07.008 15596080

[B5] Arnold H. J. Feldman D. C. (1981). Social desirability response bias in self-report choice situations. Acad. Manag. J. 24, 377–385. 10.5465/25584826914819

[B6] Arthur Jr W. Graziano W. G. (1996). The five-factor model, conscientiousness, and driving accident involvement. J. Pers. 64, 593–618. 10.1111/j.1467-6494.1996.tb00523.x 8776881

[B7] Asimakopulos J. Boychuck Z. Sondergaard D. Poulin V. Ménard I. Korner-Bitensky N. (2012). Assessing executive function in relation to fitness to drive: a review of tools and their ability to predict safe driving. Aust. Occup. Ther. J. 59, 402–427. 10.1111/j.1440-1630.2011.00963.x 23174109

[B8] Baddeley A. (1992). Working memory. Science. 255, 556–559. 10.1126/science.1736359 1736359

[B9] Bahrami B. Olsen K. Bang D. Roepstorff A. Rees G. Frith C. (2012). What failure in collective decision-making tells us about metacognition. Philos. Transact. R. Soc. B 367, 1350–1365. 10.1098/rstb.2011.0420 22492752 PMC3318766

[B10] Bahrami B. Olsen K. Latham P. E. Roepstorff A. Rees G. Frith C. D. (2010). Optimally interacting minds. Science 329, 1081–1085. 10.1126/science.1185718 20798320 PMC3371582

[B11] Beaubien J. Baker D. (2004). The use of simulation for training teamwork skills in health care: how low can you go? Qual. Saf. Health Care 13, i51–i56. 10.1136/qshc.2004.009845 15465956 PMC1765794

[B12] Bell B. S. Kozlowski S. W. (2002). A typology of virtual teams: Implications for effective leadership. Group Organ. Manag. 27, 14–49. 10.1177/1059601102027001

[B13] Blanchard M. D. Jackson S. A. Kleitman S. (2020). Collective decision making reduces metacognitive control and increases error rates, particularly for overconfident individuals. J. Behav. Decis. Mak. 33, 348–375. 10.1002/bdm.2156

[B14] Bowers C. A. Jentsch F. (2001). “Use of commercial, off-the-shelf, simulations for team research,” in Advances in Human Performance and Cognitive Engineering Research, eds Bowers C. A. Salas E. (Mahwah, NJ: Lawrence Erlbaum), 293–317.

[B15] Bowers C. A. Jentsch F. Salas E. Braun C. C. (1998). Analyzing communication sequences for team training needs assessment. Hum. Fact. 40, 672–679. 10.1518/001872098779649265

[B16] Broadway J. M. Engle R. W. (2010). Validating running memory span: measurement of working memory capacity and links with fluid intelligence. Behav. Res. Methods 42, 563–570. 10.3758/BRM.42.2.563 20479188

[B17] Bunderson J. S. Sutcliffe K. M. (2003). Management team learning orientation and business unit performance. J. Appl. Psychol. 88, 552–560. 10.1037/0021-9010.88.3.552 12814303

[B18] Carroll J. B. (1993). Human Cognitive Abilities: A Survey of Factor-Analytical Studies. New York, NY: Cambridge University Press.

[B19] Cattell R. B. (1971). Abilities: Their Structure, Growth, and Action. Boston, MA: Houghton Mifflin.

[B20] Cattell R. B. (1987). Intelligence: Its Structure, Growth and Action. Amsterdam: Elsevier.

[B21] Christensen C. Larson J. R. Abbott A. Ardolino A. Franz T. Pfeiffer C. (2000). Decision making of clinical teams: Communication patterns and diagnostic error. Med. Decis. Mak. 20, 45–50. 10.1177/0272989X0002000106 10638536

[B22] Cohen S. Cohen S. (1980). Aftereffects of stress on human performance and social behavior: a review of research and theory. Psychol. Bull. 88, 82–108. 10.1037/0033-2909.88.1.82 7403392

[B23] Cooke N. Gorman J. Duran J. Taylor A. Cooke N. (2007). Team cognition in experienced command-and-control teams. J. Exp. Psychol. Appl. 13, 146–157. 10.1037/1076-898X.13.3.146 17924800

[B24] Cooke N. J. Gorman J. C. Rowe L. J. (2009). “An ecological perspective on team cognition,” in Team Effectiveness in Complex Organizations, eds Salas E. Goodwin G. F. Burke C. S. (New York, NY: Routledge, Taylor and Francis Group), 157–182.

[B25] Cooper P. J. Zheng Y. Richard C. Vavrik J. Heinrichs B. Siegmund G. (2003). The impact of hands-free message reception/response on driving task performance. Accis. Anal. Prev. 35, 23–35. 10.1016/S0001-4575(01)00083-5 12479894

[B26] Cronbach L. J. (1951). Coefficient alpha and the internal structure of tests. Psychometrika 16, 297–334. 10.1007/BF02310555

[B27] Davidson M. C. Amso D. Anderson L. C. Diamond A. (2006). Development of cognitive control and executive functions from 4 to 13 years: Evidence from manipulations of memory, inhibition, and task switching. Neuropsychologia. 44, 2037–2078. 10.1016/j.neuropsychologia.2006.02.006 16580701 PMC1513793

[B28] De Jong R. Coles M. G. H. Logan G. D. (1995). Strategies and mechanisms in nonselective and selective inhibitory motor control. J. Exp. Psychol.: Human Percept. Perform. 21, 498–511. 10.1037/0096-1523.21.3.498 7790830

[B29] Devine D. J. Clayton L. D. Philips J. L. Dunford B. B. Melner S. B. (1999). Teams in organizations: Prevalence, characteristics, and effectiveness. Small Group Res. 30, 678–711. 10.1177/104649649903000602

[B30] Devine D. J. Philips J. L. (2001). Do smarter teams do better: A meta-analysis of cognitive ability and team performance. Small Group Res. 32, 507–532. 10.1177/104649640103200501

[B31] Diamond A. (2013). Executive functions. Annu. Rev. Psychol. 64, 135–168. 10.1146/annurev-psych-113011-143750 23020641 PMC4084861

[B32] Donchin Y. Gopher D. Olin M. Badihi Y. Biesky M. R. Sprung C. L.. (1995). A look into the nature and causes of human errors in the intensive care unit. Crit. Care Med. 23, 294–300. 10.1097/00003246-199502000-00015 7867355

[B33] Donnellan M. B. Oswald F. L. Baird B. M. Lucas R. E. (2006). The mini-IPIP scales: tiny-yet-effective measures of the Big Five factors of personality. Psychol. Assess. 18, 192. 10.1037/1040-3590.18.2.192 16768595

[B34] Driskell J. Salas E. Johnston J. (1999). Does stress lead to a loss of team perspective? Group Dyn. Theory Res. Pract. 3, 291–302. 10.1037/1089-2699.3.4.291

[B35] Driskell J. E. Johnston J. H. (1998). “Stress exposure training,” in Making Decisions Under Stress – Implications for Individual and Team Training, eds Cannon- Bowers J. A. Salas E. (Washington, DC: American Psychological Association), 191–217.

[B36] Edmunds A. Morris A. (2000). The problem of information overload in business organisations: a review of the literature. Int. J. Inf. Manage. 20, 17–28. 10.1016/S0268-4012(99)00051-1

[B37] Ellis A. (2006). System breakdown: the role of mental models and transactive memory in the relationship between acute stress and team performance. Acad. Manag. J. 49, 576–589. 10.5465/amj.2006.21794674

[B38] Endsley M. R. (1988). “Design and evaluation for situation awareness enhancement,” in Proceedings of the Human Factors Society Annual Meeting, 32 (Los Angeles, CA: Sage Publications), 97–101.

[B39] Eriksen B. A. Eriksen C. W. (1974). Effects of noise letters upon the identification of a target letter in a nonsearch task. Percept. Psychophys. 16, 143–149. 10.3758/BF03203267

[B40] Fischer U. McDonnell L. Orasanu J. (2007). Linguistic correlates of team performance: toward a tool for monitoring team functioning during space missions. Aviat. Space Environ. Med. 78, B86–B95. Available online at: https://www.ingentaconnect.com/content/asma/asem/2007/00000078/A00105s1/art00013?crawler=true&mimetype=application/pdf 17547309

[B41] Fleiss J. L. (1971). Measuring nominal scale agreement among many raters. Psychol. Bull. 76, 378. 10.1037/h0031619

[B42] Fletcher T. D. Major D. A. (2006). The effects of communication modality on performance and self-ratings of teamwork components. J. Comput. Mediat. Commun. 11, 557–576. 10.1111/j.1083-6101.2006.00027.x

[B43] Foushee H. C. (1984). Dyads and triads at 35,000 feet: factors affecting group process and aircrew performance. Am. Psychol. 39, 885–893. 10.1037/0003-066X.39.8.885

[B44] Gladstein D. Reilly N. (1985). Group decision making under threat: the tycoon game. Acad. Manag. J. 28, 613–627. 10.2307/256117

[B45] Glynn S. J. Henning R. A. (2000). “Can teams outperform individuals in a simulated dynamic control task?,” in Proceedings of the Human Factors and Ergonomics Society Annual Meeting 44 (Los Angeles, CA: SAGE Publications), 6–141.

[B46] González-Romá V. Hernández A. (2014). Climate uniformity: its influence on team communication quality, task conflict, and team performance. J. Appl. Psychol. 99, 1042–1058. 10.1037/a0037868 25198099

[B47] Gorman J. C. Cooke N. J. Amazeen P. G. (2010). Training adaptive teams. Hum. Fact. 52, 295–307. 10.1177/0018720810371689 20942257

[B48] Gorman J. C. Cooke N. J. Amazeen P. G. Fouse S. (2012). Measuring patterns in team interaction sequences using a discrete recurrence approach. Hum. Fact. 54, 503–517. 10.1177/0018720811426140 22908675

[B49] Gorman J. C. Cooke N. J. Pederson H. K. DeJoode J. A. (2005). “Coordinated awareness of situation by teams (CAST): measuring team situation awareness of a communication glitch,” in Proceedings of the Human Factors and Ergonomics Society Annual Meeting 49 (Los Angeles, CA: SAGE Publications), 274–277.

[B50] Gorman J. C. Cooke N. J. Winner J. L. (2006). Measuring team situation awareness in decentralized command and control environments. Ergonomics. 49, 1312–1325. 10.1080/00140130600612788 17008258

[B51] Haigney D. E. Taylor R. G. Westerman S. J. (2000). Concurrent mobile (cellular) phone use and driving performance: task demand characteristics and compensatory processes. Transport. Res. Part F Traffic Psychol. Behav. 3, 113–121. 10.1016/S1369-8478(00)00020-6

[B52] Helmreich R. Merritt A. Wilhelm J. (1999). The evolution of crew resource management training in commercial aviation. Int. J. Aviat. Psychol. 9, 19–32. 10.1207/s15327108ijap0901_2 11541445

[B53] Hill G. W. (1982). Group versus individual performance: Are N+1 heads better than one? Psychol. Bull. 91, 517–539. 10.1037/0033-2909.91.3.517

[B54] Hinsz V. B. Tindale R. S. Vollrath D. A. (1997). The emerging conceptualization of groups as information processors. Psychol. Bull. 121, 43–64. 10.1037/0033-2909.121.1.43 9000891

[B55] Hirst G. Mann L. (2004). A model of R and D leadership and team communication: the relationship with project performance. R D Manag. 34, 147–160. 10.1111/j.1467-9310.2004.00330.x

[B56] Ilgen D. R. Hollenbeck J. R. Johnson M. Jundt D. (2005). Teams in organizations: From input-process-output models to IMOI models. Annu. Rev. Psychol. 56, 517–543. 10.1146/annurev.psych.56.091103.070250 15709945

[B57] Jackson S. A. Kleitman S. (2014). Individual differences in decision-making and confidence: capturing decision tendencies in a fictitious medical test. Metacogn. Learn. 9, 25–49. 10.1007/s11409-013-9110-y

[B58] Jackson S. A. Kleitman S. Howie P. Stankov L. (2016). Cognitive abilities, monitoring confidence, and control thresholds explain individual differences in heuristics and biases. Front. Psychol. 7, 1559. 10.3389/fpsyg.2016.01559 27790170 PMC5062089

[B59] Jackson S. A. Kleitman S. Stankov L. Howie P. (2017). Individual differences in decision making depend on cognitive abilities, monitoring and control. J. Behav. Decis. Mak. 30, 209–223. 10.1002/bdm.1939

[B60] Kanki B. G. Helmreich R. L. Anca J. (eds.). (2010). Crew Resource Management. Kirkman, IO: Academic Press.

[B61] Keyton J. Beck S. J. Asbury M. B. (2010). Macrocognition: a communication perspective. Theoret. Iss. Ergon. Sci. 11, 272–286. 10.1080/14639221003729136

[B62] Kleitman S. Jackson S. A. Zhang L. M. Blanchard M. Rizvandi N. B. Aidman E. (2022). Applying evidence-centered design for the measurement of psychological resilience: the development and preliminary validation of a novel simulation-based assessment methodology. Front. Psychol. 12, 717568. 10.3389/fpsyg.2021.717568 35082711 PMC8786081

[B63] Koriat A. (2012). When are two heads better than one and why? Science. 336, 360–362. 10.1126/science.1216549 22517862

[B64] Koriat A. (2015). When two heads are better than one and when they can be worse: the amplification hypothesis. J. Exp. Psychol. General 144, 934. 10.1037/xge0000092 26168039

[B65] Kozlowski S. W. J. Bell B. S. (2013). “Work groups and teams in organizations,” in Handbook of Psychology: Industrial and Organizational Psychology, eds Schmitt N. W. Highhouse S. Weiner I. B. (Hoboken, NJ: John Wiley and Sons, Inc.), 412–469.

[B66] Kozlowski S. W. J. Klein K. J. (2000). “A multilevel approach to theory and research in organizations: contextual, temporal, and emergent processes,” in Multilevel Theory, Research, and Methods in Organizations: Foundations, Extensions, and New Directions, eds Klein K. J. Kozlowski S. W. J. (San Francisco, CA: Jossey-Bass), 3–90.

[B67] Krippendorff K. (2004). Measuring the reliability of qualitative text analysis data. Qual. Quantity 38, 787–800. 10.1007/s11135-004-8107-7

[B68] Laapotti S. Keskinen E. (2004). Has the difference in accident patterns between male and female drivers changed between 1984 and 2000? Accid. Anal. Prev. 36, 577–584. 10.1016/S0001-4575(03)00064-2 15094410

[B69] Lamble D. Kauranen T. Laakso M. Summala H. (1999). Cognitive load and detection thresholds in car following situations: safety implications for using mobile (cellular) telephones while driving. Accid. Anal. Prev. 31, 617–623. 10.1016/S0001-4575(99)00018-4 10487336

[B70] Langsrud Ø. (2003). ANOVA for unbalanced data: use type II instead of Type III sums of squares. Stat. Comput. 13, 163–167. 10.1023/A:1023260610025

[B71] Laughlin P. R. (2011). Social choice theory, social decision scheme theory, and group decision-making. Group Process. Intergroup Relat. 14, 63–79. 10.1177/1368430210372524

[B72] MacMillan J. Entin E. E. Serfaty D. (2004). “Communication overhead: the hidden cost of team cognition,” in Team Cognition: Understanding the Factors That Drive Process and Performance, eds Salas E. Fiore S. M. (American Psychological Association), 61–82.

[B73] Mahmoodi A. Bang D. Olsen K. Zhao Y. A. Shi Z. Broberg K.. (2015). Equality bias impairs collective decision-making across cultures. Proc. Nat. Acad. Sci. U. S. A. 112, 3835–3840. 10.1073/pnas.1421692112 25775532 PMC4378431

[B74] Marks M. A. Mathieu J. E. Zaccaro S. J. (2001). A temporally based framework and taxonomy of team processes. Acad. Mang. Rev. 26, 356–376. 10.5465/amr.2001.4845785

[B75] Marlow S. L. Lacerenza C. N. Paoletti J. Burke C. S. Salas E. (2018). Does team communication represent a one-size-fits-all approach?: A meta-analysis of team communication and performance. Organ. Behav. Human Decis. Process. 144, 145–170. 10.1016/j.obhdp.2017.08.001

[B76] Mathias J. L. Lucas L. K. (2009). Cognitive predictors of unsafe driving in older drivers: a meta-analysis. Int. Psychogeriatr. 21, 637–653. 10.1017/S1041610209009119 19470197

[B77] Mathieu J. E. Maynard M. T. Rapp T. Gilson L. (2008). Team effectiveness 1997-2007: a review of recent advancements and a glimpse into the future. J. Manage. 34, 410–476. 10.1177/0149206308316061

[B78] McDonald R. P. (1999). Test Theory: A Unified Approach. Mahwah, NJ: Erlbaum.

[B79] McKnight A. J. McKnight A. S. (1993). The effect of cellular phone use upon driver attention. Accid. Anal. Prev. 25, 259–265. 10.1016/0001-4575(93)90020-W 8323660

[B80] Mesmer-Magnus J. R. DeChurch L. A. (2009). Information sharing and team performance: a meta-analysis. J. Appl. Psychol. 94, 535–546. 10.1037/a0013773 19271807

[B81] Mislevy R. J. (2013). Evidence-centered design for simulation-based assessment. Mil. Med. 178, 107–114. 10.7205/MILMED-D-13-00213 24084311

[B82] Monsell S. (2003). Task switching. Trends Cogn. Sci. 7, 134–140. 10.1016/S1364-6613(03)00028-7 12639695

[B83] Peeters M. A. Van Tuijl H. F. Rutte C. G. Reymen I. M. (2006). Personality and team performance: a meta-analysis. Eur. J. Person. 20, 377–396. 10.1002/per.588

[B84] Pescetelli N. Rees G. Bahrami B. (2016). The perceptual and social components of metacognition. J. Exp. Psychol. Gen. 145, 949–965. 10.1037/xge0000180 27454040 PMC4961070

[B85] Pollack I. Johnson L. B. Knaff P. R. (1959). Running memory span. J. Exp. Psychol. 57, 137. 10.1037/h0046137 13641585

[B86] Predmore S. (1991). “Microcoding of communications in accident investigation: crew coordination in United 811 and United 232,” in Proceedings of the Sixth International Symposium on Aviation Psychology (Columbus, OH: Ohio State University).

[B87] Räder S. B. Henriksen A. H. Butrymovich V. Sander M. Jørgensen E. Lönn L.. (2014). A study of the effect of dyad practice versus that of individual practice on simulation-based complex skills learning and of students' perceptions of how and why dyad practice contributes to learning. Acad. Med. 89, 1287–1294. 10.1097/ACM.0000000000000373 24979287

[B88] Raven J. C. (1938–65). Progressive Matrices. New York, NY: The Psychological Corporation.

[B89] Rouse W. B. Morris N. M. (1986). On looking into the black box: prospects and limits in the search for mental models. Psychol. Bull. 100, 349. 10.1037/0033-2909.100.3.349

[B90] Sackett D. L. (1979). Bias in analytic research. J. Chronic Dis. 32, 51–63. 10.1016/0021-9681(79)90012-2 447779

[B91] Salas E. Sims D. E. Burke C. S. (2005). Is there a “big five” in teamwork? Small Group Res. 36, 555–599. 10.1177/10464964052771

[B92] Salen K. Tekinbaş K. S. Zimmerman E. (2004). Rules of Play: Game Design Fundamentals. Cambridge, MA: MIT Press.

[B93] Sarma K. M. Carey R. N. Kervick A. A. Bimpeh Y. (2013). Psychological factors associated with indices of risky, reckless and cautious driving in a national sample of drivers in the Republic of Ireland. Accid. Anal. Prev. 50, 1226–1235. 10.1016/j.aap.2012.09.020 23154054

[B94] Schneider T. R. (2004). The role of neuroticism on psychological and physiological stress responses. J. Exp. Soc. Psychol. 40, 795–804. 10.1016/j.jesp.2004.04.005

[B95] Shanks D. Brydges R. den Brok W. Nair P. Hatala R. (2013). Are two heads better than one? Comparing dyad and self-regulated learning in simulation training. Med. Educ. 47, 1215–1222. 10.1111/medu.12284 24206155

[B96] Shute V. J. Ke F. (2012). “Games, learning, and assessment,” in Assessment in Game-Based Learning: Foundations, Innovations, and Perspectives, eds Ifenthaler D. Eseryel D. Ge X.. (New York: NY, Springer), 43–58.

[B97] Smith-Jentsch K. A. (2009). “Measuring team-related cognition: The devil is in the details,” in Team Effectiveness in Complex Organizations: Cross-Disciplinary Perspectives and Approaches, eds Salas E. Goodwin G. F. Burke C. S. (Routledge/Taylor & Francis Group), 491–508.

[B98] Sniezek J. A. Henry R. A. (1989). Accuracy and confidence in group judgment. Organ. Behav. Human Decis. Process. 43, 1–28. 10.1016/0749-5978(89)90055-1

[B99] Stachowski A. A. Kaplan S. A. Waller M. J. (2009). The benefits of flexible team interaction during crises. J. Appl. Psychol. 94, 1536. 10.1037/a0016903 19916660

[B100] Stasser G. Titus W. (1985). Pooling of unshared information in group decision making: biased information sampling during discussion. J. Person. Soc. Psychol. 48, 1467. 10.1037/0022-3514.48.6.1467

[B101] Stasser G. Titus W. (1987). Effects of information load and percentage of shared information on the dissemination of unshared information during group discussion. J. Pers. Soc. Psychol. 53, 81. 10.1037/0022-3514.53.1.81

[B102] Sümer N. Lajunen T. Özkan T. (2005). “Big five personality traits as the distal predictors of road accident,” in Traffic and Transport Psychology: Theory and Application, ed Underwood G. (Oxford: Elsevier), 215–227.

[B103] Sundstrom E. McIntyre M. Halfhill T. Richards H. (2000). Work groups: From the Hawthorne studies to work teams of the 1990s and beyond. Group. Dynam. Theor. Res. Pract. 4, 44–67. 10.1037/1089-2699.4.1.44

[B104] Tabachnick B. G. Fidell L. S. Ullman J. B. (2007). Using Multivariate Statistics, Vol. 5. Boston, MA: Pearson, 481–498.

[B105] Tindale R. Kameda T. (2000). “Social sharedness” as a unifying theme for information processing in groups. Group Process. Intergr. Relat. 3, 123–140. 10.1177/1368430200003002002

[B106] Tolsgaard M. G. Madsen M. E. Ringsted C. Oxlund B. S. Oldenburg A. Sorensen J. L.. (2015). The effect of dyad versus individual simulation-based ultrasound training on skills transfer. Med. Educ. 49, 286–295. 10.1111/medu.12624 25693988 PMC5024026

[B107] Wildman J. L. Salas E. Scott C. P. (2014). Measuring cognition in teams: a cross-domain review. Hum. Fact. 56, 911–941. 10.1177/0018720813515907 25141596

[B108] Woolley A. W. Chabris C. F. Pentland A. Hashmi N. Malone T. W. (2010). Evidence for a collective intelligence factor in the performance of human groups. Science 330, 686–688. 10.1126/science.1193147 20929725

[B109] Zheng B. Jiang X. Tien G. Meneghetti A. Panton O. Atkins M. (2012). Workload assessment of surgeons: correlation between NASA TLX and blinks. Surg. Endosc. 26, 2746–2750. 10.1007/s00464-012-2268-6 22527300

