# Supplementary material for: Are two naïve and distributed heads better than one? Factors influencing the performance of teams in a challenging real-time task
Source: Front Psychol. 2023 May 12;14:1042710. doi: 10.3389/fpsyg.2023.1042710 (PMC10213526; doi:10.3389/fpsyg.2023.1042710)
Supplement: Supplementary file 1 [file Data_Sheet_1.docx]

**Supplemental Materials**

**Participant Exclusions**

A total of 22 participants (12 individuals and 5 teams) were excluded from our analyses. Eight participants (6 individuals and 1 team) were excluded because they did not complete the driving simulation. Six participants (3 teams) were excluded due to a computer error that caused one team member to observe a marginally different simulated environment from their teammate which rendered the navigator’s instructions inaccurate. Three participants (3 individuals) were excluded because they did not follow instructions during the driving simulat­­­ion. Five participants (3 individuals and 1 team) were excluded because they were identified as outliers (~4+ SDs from mean) on the dependent variables by inspecting scatterplots and computing Mahalanobis distance and Cook’s *D* for each participant (Stevens, 2002).

**Frequency Distributions for Each Condition**

**
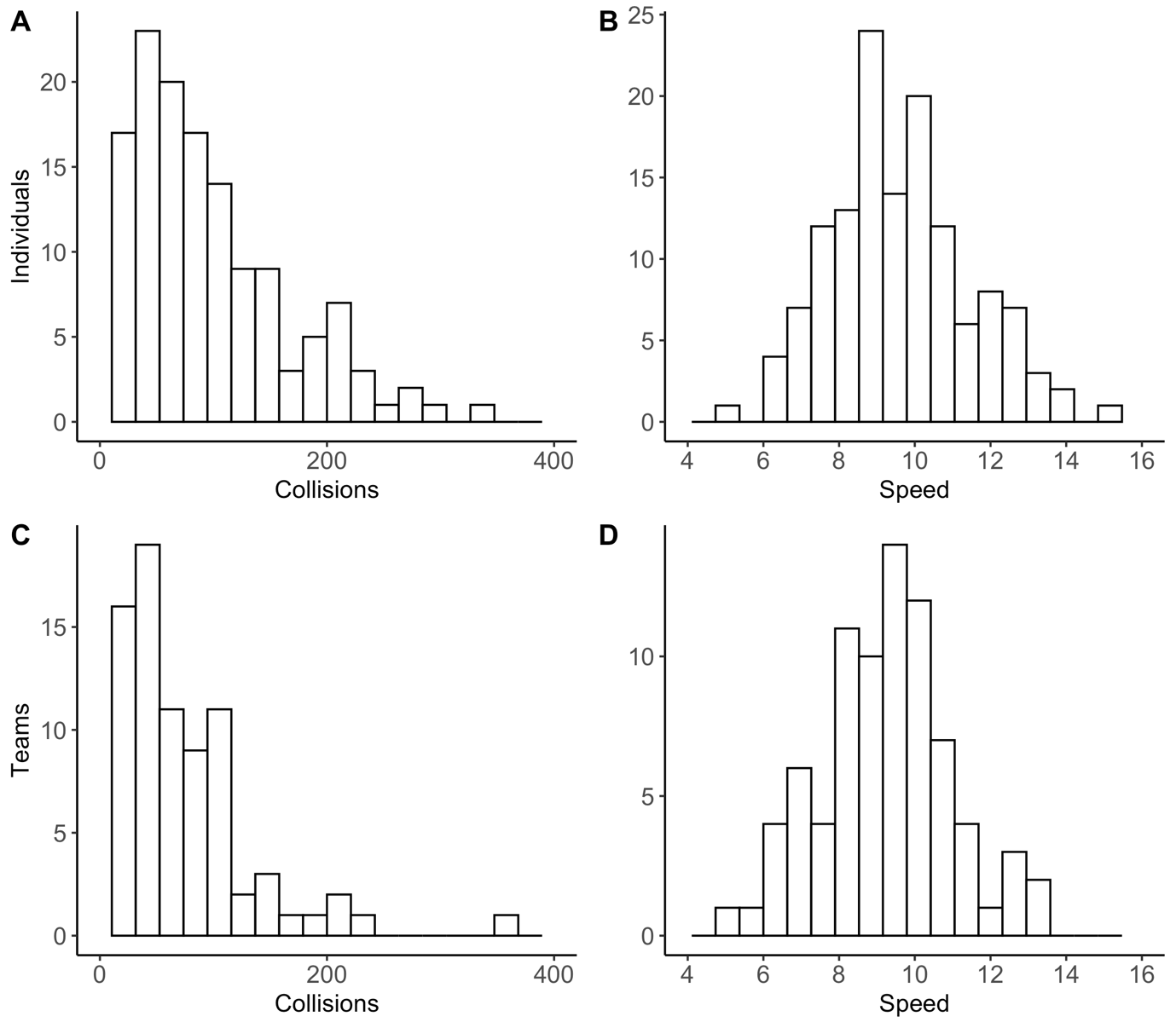
**

*Figure A1*. Frequency distributions for individual collisions (A) and speed (B) and team collisions (C) and speed (D) during the normal condition.

**
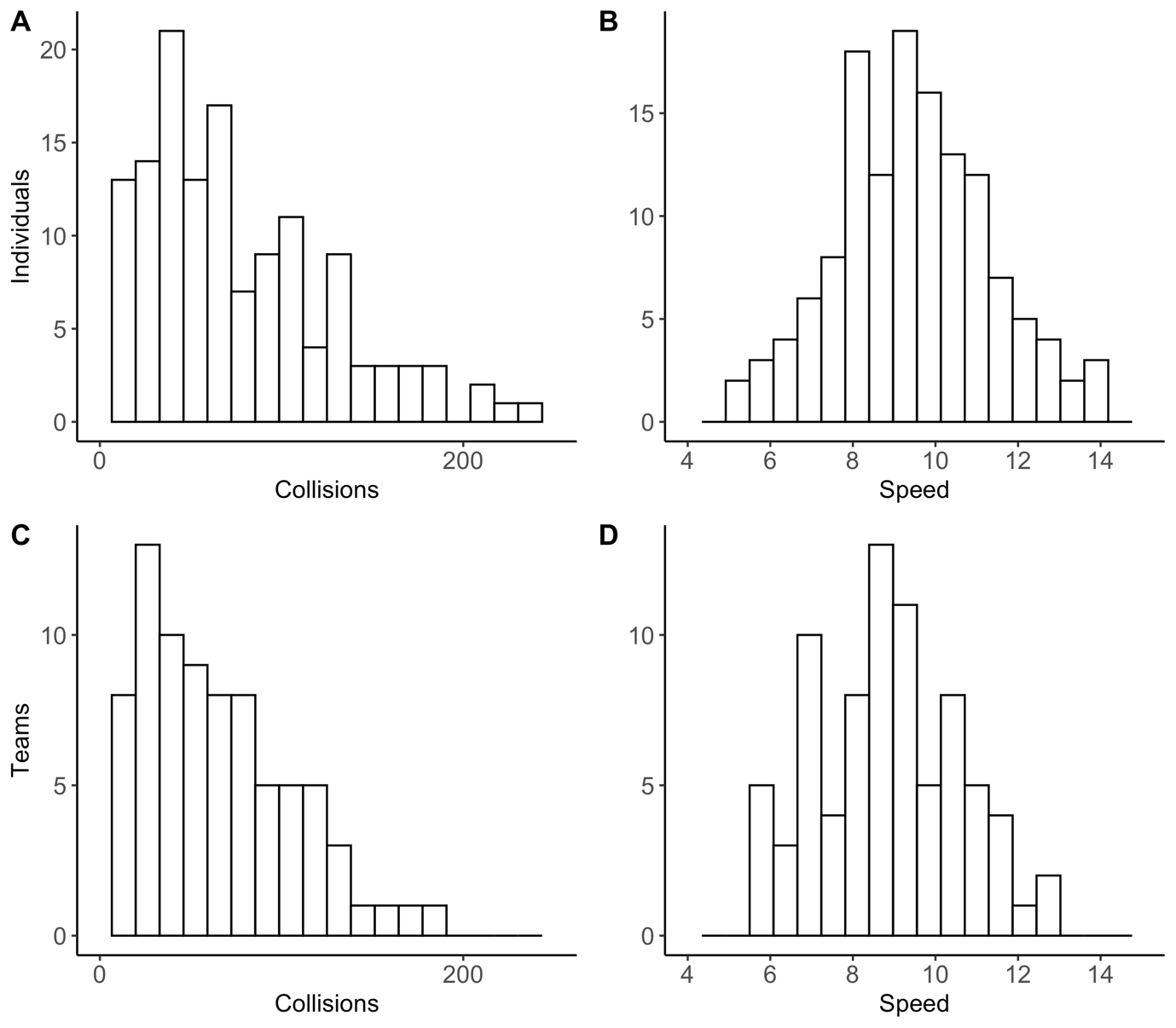
**

*Figure A2*. Frequency distributions for individual collisions (A) and speed (B) and team collisions (C) and speed (D) during the fog condition.

***Individual Differences Measures***

Descriptive statistics and internal consistency for the individual differences measures used to assess the known key covariates are presented in Table A1 for individuals and teams and Table A2 for drivers and navigators within teams.

Table A1

*Descriptive statistics for the psychometric measures and t-tests comparing individuals and teams*

|  | Individual | | |  | Team | |  |
| --- | --- | --- | --- | --- | --- | --- | --- |
|  | ω_t_ | Mean | SD | ω_t_ | Mean | SD | *t* |
| **Executive Functions** |  |  |  |  |  |  |  |
| Switching |  |  |  |  |  |  |  |
| Repeat time | .93 | 983 | 367 | .89 | 933 | 281 | 1.13 |
| Switch time | .95 | 1067 | 387 | .88 | 1042 | 306 | 0.52 |
| Repeat errors | .67 | 1.62 | 2.25 | .57 | 1.67 | 1.80 | -0.18 |
| Switch errors | .54 | 2.41 | 2.43 | .41 | 2.12 | 2.13 | 0.96 |
| Inhibition |  |  |  |  |  |  |  |
| Congruent time | .69 | 463.09 | 46.07 | .97 | 458.32 | 49.62 | 0.79 |
| Incongruent time | .82 | 531.51 | 66.78 | .84 | 530.31 | 62.61 | 0.18 |
| Congruent errors | .39 | 1.41 | 1.92 | .47 | 0.92 | 1.31 | 1.99^*^ |
| Incongruent errors | .63 | 1.66 | 2.06 | .55 | 1.52 | 1.73 | 0.50 |
| Working Memory |  |  |  |  |  |  |  |
| Accuracy | .71 | 44.78 | 18.64 | .71 | 43.04 | 18.69 | 0.72 |
| **Fluid Intelligence** |  |  |  |  |  |  |  |
| Accuracy | .81 | 53.06 | 21.33 | .82 | 57.05 | 21.38 | -1.49 |
| **Metacognition** |  |  |  |  |  |  |  |
| Trait Confidence | .92 | 61.07 | 18.21 | .92 | 61.97 | 18.05 | -0.40 |
| **Personality** |  |  |  |  |  |  |  |
| Big-Five |  |  |  |  |  |  |  |
| Agreeableness | .59 | 3.17 | 0.60 | .68 | 3.90 | 0.67 | -9.48^***^ |
| Conscientiousness | .60 | 3.08 | 0.60 | .64 | 3.29 | 0.72 | -2.63^**^ |
| Extraversion | .79 | 2.91 | 0.52 | .79 | 3.12 | 0.81 | -2.56^*^ |
| Intellect | .55 | 2.84 | 0.61 | .58 | 3.59 | 0.65 | -9.26^***^ |
| Neuroticism | .47 | 3.02 | 0.50 | .63 | 2.88 | 0.70 | 1.96 |

*Note*. IC = Internal Consistency. Internal Consistency estimates were computed using McDonald’s Omega for all variables except switching, inhibition, Bias, and discrimination. For these variables we correlated scores on the odd items with scores on the even items then adjusted the correlation coefficient using the Spearman-Brown formula (Blanchard et al., 2020; Guilford, 1954; Stankov & Crawford, 1996).
^*^*p* < .05, ^**^*p* < .01, ^***^*p* < .001

Table A2

*Descriptive statistics for the psychometric measures and t-tests comparing drivers and navigators*

|  | Driver | | | Navigator | | |  |
| --- | --- | --- | --- | --- | --- | --- | --- |
|  | ω_t_ | Mean | SD | ω_t_ | Mean | SD | *t* |
| **Executive Functions** |  |  |  |  |  |  |  |
| Switching |  |  |  |  |  |  |  |
| Repeat time | .87 | 941 | 258 | .90 | 926 | 303 | 0.33 |
| Switch time | .87 | 1040 | 291 | .88 | 1044 | 322 | -0.11 |
| Repeat errors | .29 | 1.69 | 1.59 | .69 | 1.65 | 2.01 | 0.18 |
| Switch errors | .28 | 2.08 | 1.84 | .49 | 2.16 | 2.39 | -0.27 |
| Inhibition |  |  |  |  |  |  |  |
| Congruent time | .98 | 446.41 | 47.43 | .95 | 470.39 | 49.16 | -2.82^**^ |
| Incongruent time | .87 | 515.76 | 63.06 | .80 | 545.05 | 58.96 | -2.72^**^ |
| Congruent errors | .54 | 0.95 | 1.41 | .39 | 0.90 | 1.19 | 0.31 |
| Incongruent errors | .44 | 1.49 | 1.54 | .63 | 1.56 | 1.91 | -0.22 |
| Working Memory |  |  |  |  |  |  |  |
| Accuracy | .73 | 41.67 | 19.46 | .68 | 44.42 | 17.91 | -0.98 |
| **Fluid Intelligence** |  |  |  |  |  |  |  |
| Accuracy | .82 | 56.39 | 21.08 | .83 | 57.71 | 21.79 | -0.37 |
| **Metacognition** |  |  |  |  |  |  |  |
| Trait Confidence | .93 | 62.01 | 18.09 | .92 | 61.94 | 18.13 | 0.02 |
| **Personality** |  |  |  |  |  |  |  |
| Big-Five |  |  |  |  |  |  |  |
| Agreeableness | .71 | 3.95 | 0.67 | .64 | 3.85 | 0.68 | 0.91 |
| Conscientiousness | .69 | 3.18 | 0.74 | .56 | 3.40 | 0.68 | -1.80 |
| Extraversion | .83 | 3.10 | 0.85 | .74 | 3.14 | 0.78 | -0.39 |
| Intellect | .53 | 3.58 | 0.65 | .63 | 3.60 | 0.67 | -0.23 |
| Neuroticism | .64 | 2.92 | 0.73 | .62 | 2.84 | 0.66 | 0.72 |

*Note*. IC = Internal Consistency. Internal Consistency estimates were computed using McDonald’s Omega for all variables except switching, inhibition, Bias, and discrimination. For these variables we correlated scores on the odd items with scores on the even items then adjusted the correlation coefficient using the Spearman-Brown formula (Blanchard et al., 2020; Guilford, 1954; Stankov & Crawford, 1996).
^**^*p* < .01, ^***^*p* < .001

Individuals and teams significantly differed on each of the personality facets except Neuroticism. Individuals were lower on Agreeableness, Conscientiousness, Extraversion, and Intellect compared with teams. Within teams, drivers were significantly faster than navigators on congruent time and incongruent time. No other significant differences were observed. All reliability estimates were acceptable for research purposes except repeat errors (team *a* = .57 and driver *a* = .29), switch errors (all levels, *a* = .28 - .54), congruent errors (all levels, *a* = .39 - .54), incongruent errors (team *a* = .55 and driver *a* = .44), conscientiousness (codriver *a* = .56), intellect (individual = .55, team *a* = .58, driver *a* = .53), and neuroticism (individual *a* = .47) which ranged from low to poor. The repeat errors, switch errors, congruent errors, and incongruent errors variables consistently demonstrated poor internal consistency thus they were removed from subsequent analyses. These four variables assessed inhibitory control and cognitive flexibility. We had two different metrics for each of these constructs: errors and response time. The response time measures demonstrated excellent reliability (ranging from ω_t_ = .88 - .95), thus, they remained in the study for our analyses. Reliability estimates for the personality measures ranged between ω_t_ = .47 - .79 for individuals and ω_t_ = .58 - .79 for teams. Some of the reliability estimates for individuals were low, however, we only used the team measures as control variables to examine hypotheses related to aims 2 and 3. Overall, these estimates were consistent with previous literature using this brief instrument (Blanchard et al., 2020; Jackson et al., 2016; Jackson et al., 2017).

**Reduction of independent variables**

To retain adequate power in our hierarchical regression analyses for hypotheses 2 and 3, we reduced the independent variables for teams down to a smaller number of components using PCA. These extracted components were: Executive Function Time which was composed of the response time variables for repeat time, switch time, congruent time, and incongruent time; and Competence which was composed of fluid intelligence, confidence, and working memory accuracy. These PCAs and the correlations between all outcome variables, team composition measures, and control variables are presented below.

Table B1

*Intercorrelations and PCA results for Executive Function time variables*

|  | Pearson *r* correlations | | | Component loadings | |
| --- | --- | --- | --- | --- | --- |
|  | 2 | 3 | 4 | 1 | *h^2^* |
| 1. Switch time | .89 | .45 | .34 | **.84** | .70 |
| 2. Repeat time |  | .40 | .29 | **.81** | .65 |
| 3. Congruent time |  |  | .84 | **.83** | .69 |
| 4. Incongruent time |  |  |  | **.75** | .57 |

*Note*. D=Driver; N=Navigator; EF variables = Executive Function variables; Component loadings >.30 are in bold. *h^2^*= communality.

^***^*p* < .001, ^**^*p* < .01, ^*^*p* < .05

First, we extracted latent component(s) of Executive Function time. The correlations between these variables and a summary of the results of the PCA are presented in Table B1.

A pattern of small to large positive correlations was evident between all variables. We conducted a PCA (with Promax rotation) on repeat time, switch time, congruent time, and incongruent time. Inspection of scree plots, the Kaiser criterion, and Horn’s Parallel Analysis (with 5000 iterations: Horn, 1965) suggested a two-component solution. However, to reduce the number of variables to include in the final model we extracted a single component which explained 65% of the common variance.

All time variables loaded positively on this component which was named EF Time.

Table B2

*Intercorrelations and PCA results for competence variables*

|  | Pearson *r* correlations | | Component loadings | |
| --- | --- | --- | --- | --- |
|  | 2 | 3 | 1 | *h^2^* |
| 1. Fluid intelligence | 0.40 | 0.35 | **.72** | .52 |
| 2. Confidence | 1 | 0.20 | **.82** | .67 |
| 3. Working memory |  | 1 | **.67** | .45 |

*Note*. D=Driver; N=Navigator; Component loadings >.30 are in bold. *h^2^*= communality.

^***^*p* < .001, ^**^*p* < .01, ^*^*p* < .05

Next, we extracted latent component(s) of Competence. The correlations between these variables and a summary of the results of the PCA are presented in Table B2.

A pattern of small to moderate positive correlations was evident between all variables. We conducted a PCA (with Promax rotation) on fluid intelligence, confidence, and working memory accuracy. Inspection of scree plots, the Kaiser criterion, and Horn’s Parallel Analysis (with 5000 iterations: Horn, 1965) suggested a one-component solution. We extracted a single component which explained 55% of the common variance. All time variables loaded positively on this component which was named Competence.

The EF time and Competence components were included as control variables in the hierarchical regression analyses related to hypotheses 2 and 3.
